# Nanomineral-fueled chemolithoautotrophy leads to substantial mercury emission

**DOI:** 10.1093/nsr/nwaf581

**Published:** 2025-12-19

**Authors:** Zeyou Chen, Chenyang Zhang, Zhanhua Zhang, Qing Chang, Cheng Gao, Xiao Liang, Xin Nie, Long Chen, Zhi Cao, Yan Lin, Pedro J J Alvarez, Wei Chen, Tong Zhang, Cong-Qiang Liu

**Affiliations:** College of Environmental Science and Engineering, Ministry of Education Key Laboratory of Pollution Processes and Environmental Criteria, Tianjin Key Laboratory of Environmental Remediation and Pollution Control, Nankai University, Tianjin 300350, China; College of Environmental Science and Engineering, Ministry of Education Key Laboratory of Pollution Processes and Environmental Criteria, Tianjin Key Laboratory of Environmental Remediation and Pollution Control, Nankai University, Tianjin 300350, China; Agro-Environmental Protection Institute, Ministry of Agriculture and Rural Affairs, Tianjin 300191, China; College of Environmental Science and Engineering, Ministry of Education Key Laboratory of Pollution Processes and Environmental Criteria, Tianjin Key Laboratory of Environmental Remediation and Pollution Control, Nankai University, Tianjin 300350, China; College of Environmental Science and Engineering, Ministry of Education Key Laboratory of Pollution Processes and Environmental Criteria, Tianjin Key Laboratory of Environmental Remediation and Pollution Control, Nankai University, Tianjin 300350, China; College of Environmental Science and Engineering, Ministry of Education Key Laboratory of Pollution Processes and Environmental Criteria, Tianjin Key Laboratory of Environmental Remediation and Pollution Control, Nankai University, Tianjin 300350, China; College of Environmental Science and Engineering, Ministry of Education Key Laboratory of Pollution Processes and Environmental Criteria, Tianjin Key Laboratory of Environmental Remediation and Pollution Control, Nankai University, Tianjin 300350, China; School of Geographic Sciences, East China Normal University, Shanghai 200241, China; College of Environmental Science and Engineering, Ministry of Education Key Laboratory of Pollution Processes and Environmental Criteria, Tianjin Key Laboratory of Environmental Remediation and Pollution Control, Nankai University, Tianjin 300350, China; College of Environmental Science and Engineering, Ministry of Education Key Laboratory of Pollution Processes and Environmental Criteria, Tianjin Key Laboratory of Environmental Remediation and Pollution Control, Nankai University, Tianjin 300350, China; Department of Civil and Environmental Engineering, Rice University, Houston, TX 77005, USA; College of Environmental Science and Engineering, Ministry of Education Key Laboratory of Pollution Processes and Environmental Criteria, Tianjin Key Laboratory of Environmental Remediation and Pollution Control, Nankai University, Tianjin 300350, China; College of Environmental Science and Engineering, Ministry of Education Key Laboratory of Pollution Processes and Environmental Criteria, Tianjin Key Laboratory of Environmental Remediation and Pollution Control, Nankai University, Tianjin 300350, China; School of Earth System Science, Tianjin University, Tianjin 300072, China

**Keywords:** HgS nanoparticles, mercury emission, microbial transformation, chemolithoautotrophic bacteria

## Abstract

Elemental mercury (Hg^0^) is a pervasive pollutant of global concern. Recent research shows that global Hg^0^ emissions are significantly underestimated, but the additional sources contributing to this gap are unclear. Here, we demonstrate that chemolithoautotrophic microorganisms, such as sulfur-oxidizing and iron-oxidizing bacteria that are ubiquitous in diverse environments, utilize mercury sulfide (HgS) nanoparticles to support growth while releasing substantial amounts of Hg^0^. Unlike dissolved Hg(II) species, HgS nanoparticles (HgS_NP_) are internalized into bacterial cells through an adenosine triphosphate (ATP)-independent, highly energy-efficient process. This enhanced internalization leads to rapid metabolism of HgS_NP_ via sulfur-oxidation, as well as the subsequent reduction of the released Hg(II) to Hg^0^ mediated by the *mer* operon, superoxide or cytochrome *c*. Our modeling results show that the annual emission flux of Hg^0^ from this previously unrecognized source can reach 272.44 ± 134.99 tonnes, equivalent to that of geogenic Hg^0^ emissions and matching that of the fourth largest anthropogenic source, cement production.

## Teaser

Chemolithoautotrophic bacteria tap into nanomineral stores of mercury sulfide, converting these particles into gaseous mercury. This study reveals a previously overlooked natural pathway that releases mercury to the atmosphere and quantifies its global significance for the first time.

## INTRODUCTION

Elemental mercury (Hg^0^) is a pervasive pollutant of global concern, due to its ability to vaporize at ambient temperatures and to undergo long-distance atmospheric transport [1–4]. Hg^0^ is released through both natural processes (e.g. volcanic eruptions, geothermal activities and rock weathering) and anthropogenic activities (e.g. gold mining, coal combustion and industrial operations) [5–11]. Since the Minamata Convention came into effect in 2017, global initiatives have been taken to identify and control the anthropogenic sources of mercury, which account for approximately 30% of the total global mercury emissions [5,12–14]. In contrast, the processes driving natural emission of deposited Hg from the earth’s crust, which contribute to over 60% of Hg^0^ release [3,10,15], remain understudied. For instance, a recently developed atmospheric–terrestrial–oceanic coupled model reveals that global Hg^0^ emissions may be underestimated by as much as 40% [16]. Predictions based on global mercury isotope box models also indicate that the standard rate constants used in global mercury cycling models significantly underestimate Hg^0^ emissions [17]. Thus, identifying additional sources of natural Hg^0^ release and understanding mechanisms controlling these processes are of critical importance for improving the accuracy of global Hg emission inventories, and for advancing mercury pollution control measures.

Both abiotic and biotic processes contribute to Hg^0^ formation in natural environments [18–22]. Photochemical reduction of dissolved Hg(II) species by UV light has been recognized as the key abiotic process producing Hg^0^ in surface waters, surface soils and atmospheric aerosols [18–20]. For biotic sources of Hg^0^, microbial mercury reduction driven primarily by the *mer* operon is the most widely recognized pathway [22–24]. It is necessary to note that the majority of the earth’s habitats exist in dark conditions [25], and Hg levels in natural environments are often too low to activate the *mer* operon, which functions as a defense mechanism against high mercury exposure [26,27]. Nonetheless, mounting evidence indicates the existence of exceptionally high levels of Hg^0^ in the world’s deepest marine environments such as trench areas and deep-sea cold seeps, wherein dissolved Hg(II) levels are low and light is absent [28–31]. This points to the undiscovered additional mechanisms driving Hg^0^ production in natural environments.

Previous studies show that autotrophic bacteria may use HgS minerals as natural habitats [32–34]. For example, incubating HgS minerals near a diffuse-flow hydrothermal vent on the East Pacific Rise resulted in colonization of sulfur-oxidizing bacteria at relatively high abundance [34]. Similarly, sulfur-oxidizing bacteria of the genus *Thiobacillus* were enriched on metacinnabar chips incubated in creek sediments, and laboratory incubation of these mineral samples led to release of Hg(II) and Hg^0^, though at particularly slow rates [33]. Note that these mercury transformation processes may become dominant when the microbe–mineral interactions occur at nanoscale, since mineral nanoparticles are a major mercury species at microbial habitats [35–37] and nanoparticles often exhibit unique environmental behaviors differing from their dissolved and bulk-scale counterparts [33,38–42]. However, the fundamental processes governing Hg^0^ formation from HgS nanoparticles (HgS_NP_) by autotrophic bacteria remain poorly understood and unquantified. Indeed, our research reports direct evidence that chemolithoautotrophy on HgS_NP_ is a previously unrecognized and yet essential source of Hg^0^. Chemolithoautotrophic bacteria internalize HgS_NP_ through an adenosine triphosphate (ATP)-independent process, and metabolize reduced sulfur while releasing Hg(II). In response, a diverse variety of reduction pathways are triggered to generate substantial amount of Hg^0^.

## RESULTS AND DISCUSSION

### HgS_NP_ support chemolithoautotrophy while producing Hg^0^

**Figure 1.**
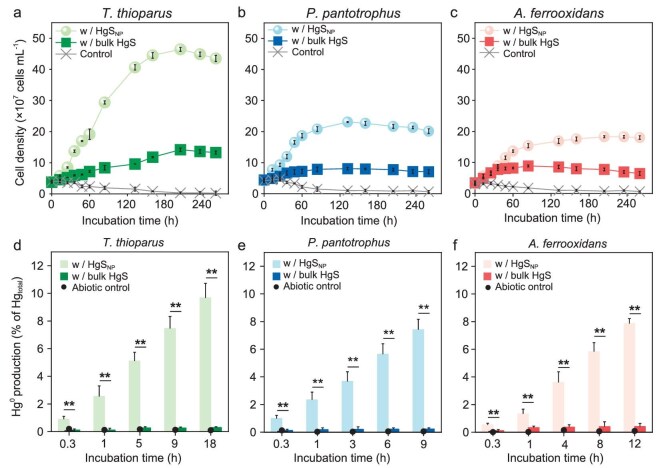
Substantial Hg^0^ production during growth of chemolithoautotrophic bacteria upon exposure to HgS_NP_. (a–c) Growth curves of *T. thioparus* (a), *P. pantotrophus* (b) and *A. ferrooxidans* (c) cells upon exposure to 50 μM HgS_NP_ or bulk HgS. Control group represents culture media without mercury addition. (d–f) Hg^0^ production by *T. thioparus* (d), *P. pantotrophus* (e) and *A. ferrooxidans* (f) cells upon exposure to 50 μM HgS_NP_ or bulk HgS. Control represents cultures without mercury addition. Abiotic control represents the uninoculated media with HgS_NP_. Error bars represent ±1 SD of triplicates. Statistically different values (*P* < 0.01) are indicated by asterisks (**) according to an independent two-sample *t*-test.

Chemolithoautotrophic bacteria, including the archetypes for sulfur-oxidizing bacteria *Thiobacillus thioparus* (ATCC 8158) and *Paracoccus pantotrophus* (ATCC 35512), and iron-oxidizing bacteria *Acidithiobacillus ferrooxidans* (ATCC 23270), were able to use HgS_NP_ as growth substances and consequently, produced substantial quantities of Hg^0^ during chemolithoautotrophy, with transformation efficiencies of 9.7% ± 1.1%, 7.4% ± 0.8% and 7.9% ± 0.4% of total mercury for *T. thioparus, P. pantotrophus* and *A. ferrooxidans*, respectively. When HgS_NP_ were incubated in the culture media as the sole electron donors, cell density of *T. thioparus* increased rapidly for approximately 160 h, and the growth rate markedly surpassed that of the treatment with bulk-sized HgS (Fig. 1a). Less significant growth was observed for *P. pantotrophus* (Fig. 1b) and *A. ferrooxidans* (Fig. 1c), but the overall trend was consistent, i.e. HgS_NP_ sustained cell growth to a higher extent than its bulk-sized counterpart (Fig. 1a–c). More interestingly, production of Hg^0^ from HgS_NP_ increased appreciably with time, whereas Hg^0^ production from bulk-sized HgS by all bacterial strains was minimal and increased little with time (Fig. 1d–f). In *T. thioparus*, microbe–nanoparticle interactions led to the rapid reduction of up to 9.7% ± 1.1% of total mercury to elemental Hg^0^ during the early exponential growth phase (0–18 h, Fig. 1a and d). Clearly, different mechanisms were in play in the microbial transformation of HgS_NP_, as compared to bulk minerals.

**Figure 2.**
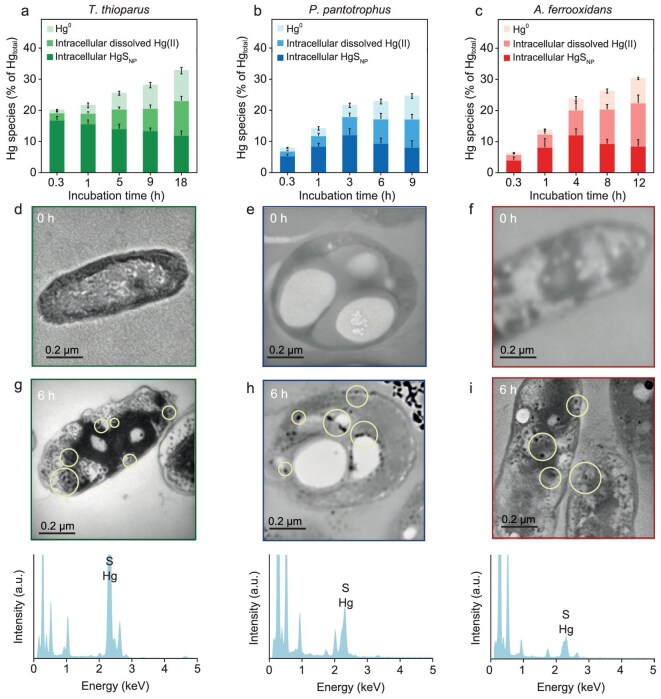
Hg^0^ release is accompanied by intracellular uptake and dissolution of HgS_NP_. (a–c) Comparison of mass fractions of Hg^0^, intracellular dissolved Hg(II), and intracellular HgS_NP_ in *T. thioparus* (a), *P. pantotrophus* (b) and *A. ferrooxidans* (c) cells upon exposure to 50 μM HgS_NP_. (d–i) TEM images of thin section of *T. thioparus* (d and g), *P. pantotrophus* (e and h) and *A. ferrooxidans* (f and i) cells before (d–f) and after 6-h exposure (g–i) to 50 μM HgS_NP_, and EDX spectra of intracellular HgS_NP_. Error bars represent ±1 SD of triplicates.

To understand the mechanisms facilitating the transformation of HgS_NP_ into Hg^0^, we conducted mercury fractionation analysis, including intracellular nanoparticulate mercury, intracellular dissolved mercury and evaporable mercury, during the microbe–nanoparticle interactions (Fig. 2 and Fig. S8). Interestingly, the fraction of intracellular nanoparticles appeared immediately, and at 0.3 h the content of HgS_NP_ in *T. thioparus* accounted for 16.8% ± 1.2% of the total mercury mass initially added (Fig. 2a). Substantial occurrence of nanoparticulate mercury was also observed for *P. pantotrophus* and *A. ferrooxidans* (Fig. 2b and c). Transmission electron microscopy (TEM) coupled with energy-dispersive X-ray spectroscopy (EDX) analysis showed that these nanoparticles were mainly comprised of Hg and S elements and located in the cell cytoplasm (Fig. 2d–i), indicating the direct uptake of HgS_NP_ by the bacteria. Moreover, the time-dependent increase in the concentrations of intracellular dissolved mercury (Fig. 2a–c) suggests that the internalized HgS_NP_ were readily dissolved. In contrast, when bulk-sized HgS was incubated with the bacteria, the intracellular mercury fraction was primarily dissolved species and the abundance was extremely low (Figs S4 and S7), demonstrating that the microbial uptake and dissolution of bulk-sized HgS were minimal. While internalization of nanoparticles into bacterial cells has been reported, this process is typically associated with cell membrane damage [43–45], yet the integrity of cell membranes of all chemolithoautotrophic bacteria in our experiments did not appear to be compromised by interactions with HgS_NP_ (Fig. S5), which infers the existence of a highly efficient pathway for microbial uptake of nanoparticulate mercury.

### ATP-independent transmembrane transport of HgS_NP_

**Figure 3.**
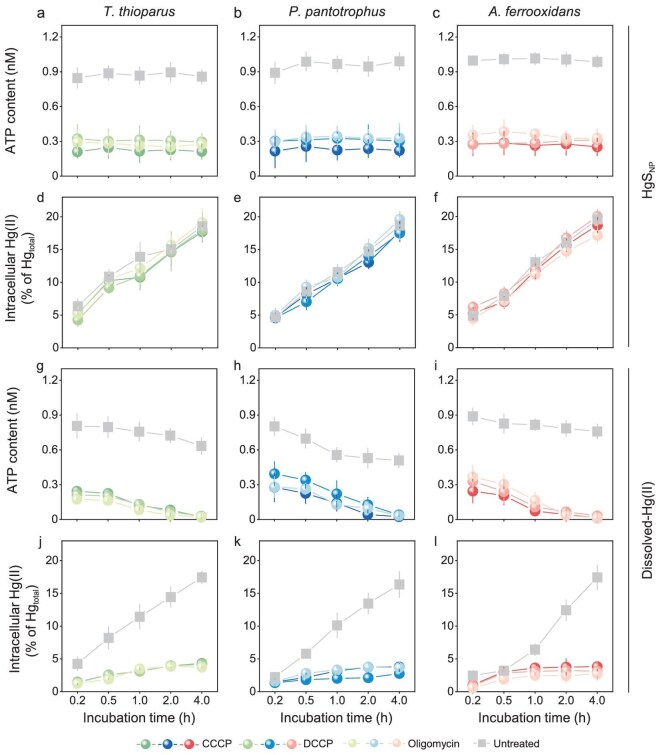
ATP-independent cellular uptake of HgS_NP_. (a–f) Significant decrease in ATP contents (a–c) in *T. thioparus* (a and d), *P. pantotrophus* (b and e) and *A. ferrooxidans* (c and f) cells upon exposure to 50 μM HgS_NP_ in the presence of 5 μM proton uncoupler CCCP, 10 μM ATP synthase inhibitors DCCD and oligomycin did not affect intracellular Hg(II) in bacteria (d–f), showing the uptake of HgS_NP_ is an ATP-independent passive transport process. In contrast (g–l), significant decrease in ATP contents (g–i) in *T. thioparus* (g and j), *P. pantotrophus* (h and k) and *A. ferrooxidans* (i and l) cells upon exposure to 50 μM dissolved Hg(II) in the presence of 5 μM CCCP, 10 μM DCCD and 10 μM oligomycin substantially decreased intracellular Hg(II) in bacteria (j–l), showing the uptake of dissolved Hg(II) by bacteria is an ATP-dependent transport process. Error bars represent ±1 SD of triplicates.

**Figure 4.**
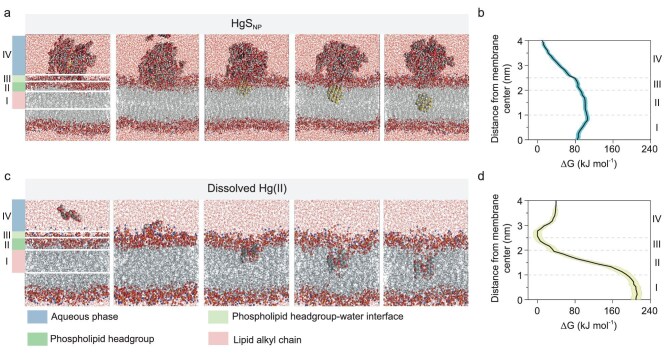
Molecular dynamic simulations showing HgS_NP_ more easily penetrates cell membranes than dissolved Hg(II) due to its lower free energy barrier for transmembrane processes. (a and c) Snapshots of HgS_NP_ (a) and dissolved Hg(II) (c) penetrating the phospholipid bilayer of cell membranes during the simulations. (b and d) Gibbs free energy changes (ΔG) along the penetration processes of HgS_NP_ (b) and dissolved Hg(II) (d). Region I is the lipid alkyl chain (z = 0–1 nm). Region II is the phospholipid headgroup, which is the hydrophobic–hydrophilic interface (z = 1–2 nm). Region III is the phospholipid headgroup–water interface (z = 2–2.5 nm). Region IV is the aqueous phase (z > 2.5 nm). Shaded areas represent ±1 SD of triplicates.

HgS_NP_ were internalized into the cells through an ATP-independent, passive diffusion fashion (Figs 3 and 4). When exposed to HgS_NP_, the ATP content of the bacteria remained relatively stable with increasing incubation time (Fig. 3a–c), whereas a continuous decrease in ATP levels was observed when the bacteria were exposed to dissolved Hg(II) (Fig. 3g–i). Apparently, cellular uptake of HgS_NP_ did not require ATP as the key energy carrier, as in the case of dissolved Hg(II). To test this hypothesis, we inhibited ATP production by the bacteria using a proton uncoupler carbonyl cyanide *m*-chlorophenyl hydrazine (CCCP) and two ATP synthase inhibitors *N, N′*-dicyclohexylcarbodiimide (DCCD) and oligomycin [46,47]. Consistent with our hypothesis, the decreased intracellular ATP levels in the bacteria resulted in significantly inhibited cellular uptake of dissolved Hg(II) (Fig. 3g–l), in line with the well-acknowledged mechanism of active transport of Hg(II) that consumes ATP [48,49]. Conversely, the decreased ATP levels had no effects on the uptake of HgS_NP_ (Fig. 3a–f). Additionally, no significant membrane damage or acute cytotoxicity was observed under the tested conditions (Figs S9 and S10), indicating that HgS_NP_ uptake was not due to compromised membrane integrity. These results indicate that cross-membrane transport of HgS_NP_ likely occurred through passive diffusion.

Previous studies showed that nanoparticle internalization is strongly size-dependent [50,51]. Accordingly, we used 4–5 nm HgS_NP_ (Fig. S1) to match the sizes of primary particles identified in laboratory studies [36,40–42,52–54]. To evaluate the thermodynamic feasibility of transmembrane transport and to identify the rate-limiting step, we performed molecular dynamic simulations to quantify the energy landscape associated with membrane crossing by HgS_NP_ within this relevant size range, and compared it with that of dissolved Hg(II) species (natural organic matter was included to better approximate environmentally realistic conditions [55]) (Fig. 4). The energy barriers were calculated across four regions (Fig. 4a and c): (i) the transition from the bulk aqueous phase to the outer membrane (region VI); (ii) passage through the water–phospholipid headgroup interface (region III), (iii) movement from the phospholipid headgroups to the hydrophobic–hydrophilic interface (region II); and (iv) entry from the phospholipid headgroups into the lipid alkyl chains (region I). For the HgS_NP_, the energy barrier is highest in region I, near the interface with region II, with a Gibbs free energy change (ΔG) value of 105 kJ/mol (Fig. 4b). Notably, this energy barrier is substantially lower than that of dissolved Hg(II), with a ΔG value reaching 212 kJ/mol in region I (Fig. 4d). This significantly lower energy barrier indicates that passive transmembrane transport is thermodynamically far more feasible for the nanoparticle than for the dissolved species. Based on these ΔG values, the estimated cross-membrane rate of HgS_NP_ is 5.6 × 10^18^ times higher than that of dissolved species (for which passive diffusion is essentially not viable). These theoretical calculations demonstrate that the key limiting factor for HgS_NP_ transport is the energy barrier for membrane translocation, which is surmountable via passive diffusion of the nanoparticles. This theoretical result corroborates the experimentally observed rapid internalization of HgS_NP_, which enabled ready transformation to release dissolved Hg(II) (Fig. 2).

### Intracellular transformation of HgS_NP_

**Figure 5.**
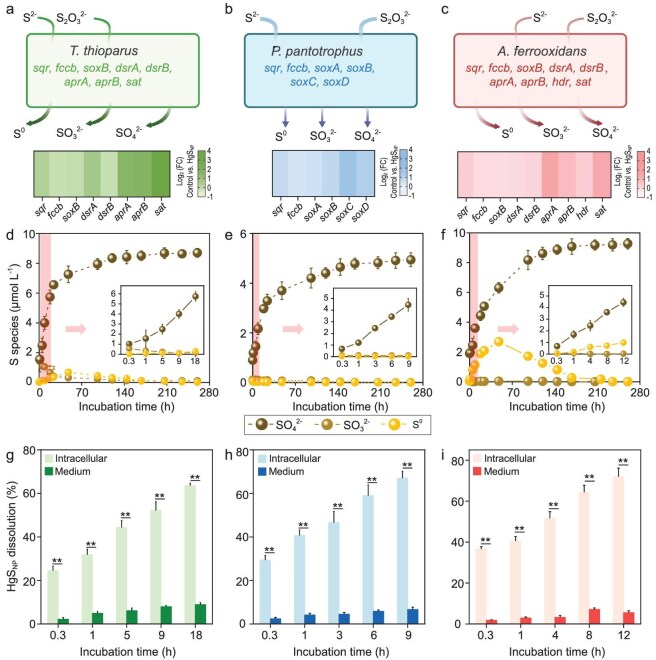
Chemolithoautotrophic bacteria utilize reduced sulfur from HgS_NP_ for growth, which induces intracellular dissolution of HgS_NP_. (a–c) Expression of genes related to sulfide oxidation in *T. thioparus* (a), *P. pantotrophus* (b) and *A. ferrooxidans* (c) cells upon exposure to 50 μM HgS_NP_. (d–f) Concentrations of SO_4_^2–^, SO_3_^2–^ and S^0^ produced by *T. thioparus* (d), *P. pantotrophus* (e) and *A. ferrooxidans* (f) cells upon exposure to 50 μM HgS_NP_. (g–i) Dissolution of HgS_NP_ in the absence and presence of *T. thioparus* (g), *P. pantotrophus* (h) and *A. ferrooxidans* (i) cells upon exposure to 50 μM HgS_NP_. Error bars represent ±1 SD of triplicates. Statistically different values (*P* < 0.01) are indicated by asterisks (**) according to an independent two-sample *t*-test.

Upon uptake of HgS_NP_, oxidative metabolism of sulfide was quickly initiated by the cells, resulting in continuous production of higher-valence sulfur species, which coincided with the release of dissolved Hg(II) (Fig. 5). Significant upregulations of sulfur oxidation-related genes were observed in all chemolithoautotrophic bacteria exposed to HgS_NP_, although different metabolic genes were involved for each strain (Fig. 5a–c). The gene with the highest upregulated expression was *sat* in *T. thioparus, soxC* in *P. pantotrophus* and *aprA* in *A. ferrooxidans* (Fig. 5a–c). The differential expression patterns of the metabolic genes across different bacteria were consistent with previous observations [56–58], and suggests that the ability of internalizing and metabolizing HgS_NP_ may distribute across microbial strains inhabiting distinct ecological niches and harbor different sulfur metabolism pathways.

Three oxidation products of sulfide were observed during microbe–nanoparticle interactions: SO_4_^2−^, S_2_O_3_^2−^ and S^0^, with SO_4_^2−^ being the most dominant product. The accumulation of SO_4_^2−^ occurred over the entire duration of bacterial growth (Fig. 5d–f), indicating that reduced sulfur from HgS_NP_ was used as the electron donors by the chemolithoautotrophic bacteria. Moreover, the fast SO_4_^2−^ formation in the initial stage of HgS_NP_ transformation (i.e. the insets of Fig. 5d–f) appeared simultaneously with the dissolution of HgS_NP_ (Fig. 5g–i). These consistent trends further demonstrate that the sulfide being metabolized and the Hg(II) being reduced inside bacterial cells likely originated from the same precursor compounds. Overall, it can be concluded that the rapid intracellular oxidation of sulfide provided an energy source to sustain chemolithoautotrophy, and this process resulted in the concurrent intracellular accumulation of dissolved Hg(II).

### Mercury detoxification by chemolithoautotrophic bacteria

**Figure 6.**
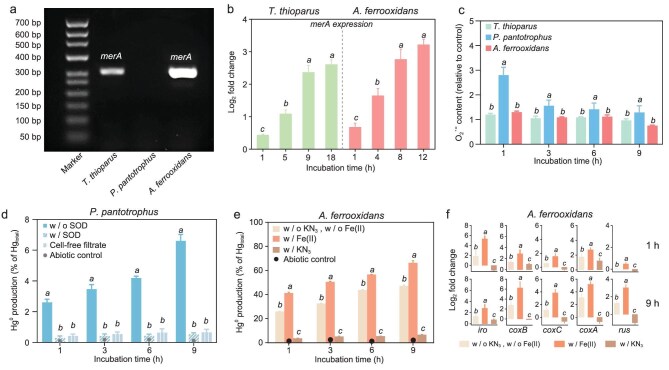
Elemental mercury is released as the result of microbial detoxification through multiple intracellular reduction processes. (a) Agarose gel electrophoresis showing *merA* gene is present in *T. thioparus* and *A. ferrooxidans* genomes, but absent in the *P. pantotrophus* genome. (b) Expression of *merA* gene in *T. thioparus* and *A. ferrooxidans* cells upon exposure to 50 μM HgS_NP_, showing the presence of an intracellular mercury reduction pathway mediated by the *merA* gene in *T. thioparus* and *A. ferrooxidans*. (c) O_2_^•–^ produced by *T. thioparus, P. pantotrophus* and *A. ferrooxidans* cells upon exposure to 50 μM HgS_NP_. (d) Hg^0^ production by *P. pantotrophus* cells upon exposure to 50 μM HgS_NP_ in the absence and presence of SOD, showing O_2_^•–^ plays an important role in the reduction of mercury in *P. pantotrophus*. (e and f) Hg^0^ production by *A. ferrooxidans* (e) and the expressions of genes (f) encoding iron oxidase, ceruloplasmin and cytochrome *c* oxidase in *A. ferrooxidans* cells upon exposure to dissolved Hg(II) in the absence of KN_3_ and Fe(II), presence of Fe(II), and presence of KN_3_, showing the presence of a membrane mercury reduction pathway mediated by cytochrome *c* oxidase in *A. ferrooxidans*. Abiotic control represents the uninoculated media. Error bars represent ±1 SD of triplicates. Statistically different values (*P* < 0.05) are indicated by lowercase letters according to the one-way analysis of variance (ANOVA). Statistically different values (*P* < 0.01) are indicated by asterisks (**) according to an independent two-sample *t*-test.

The Hg(II) released due to intracellular dissolution of HgS_NP_ was reduced to Hg^0^ by the bacteria via different detoxification mechanisms that likely facilitated sustained bacterial uptake and transformation of HgS_NP_. Polymerase chain reaction (PCR) amplification data showed that a 285 bp PCR product was amplified in the genomes of *T. thioparus* and *A. ferrooxidans*, but not in *P. pantotrophus*, and sequencing confirmed this product as the *merA* gene (Fig. 6a). Furthermore, real-time quantitative PCR (RT-qPCR) results showed that the expression *of* the *merA* genes increased over time in response to HgS_NP_ exposure (Fig. 6b), indicating that *merA*-mediated intracellular mercury reduction played an essential role in the conversion of HgS_NP_ to Hg^0^ by *T. thioparus* and *A. ferrooxidans*. These were in line with the widely accepted knowledge that the *mer* operon system existing in a diverse variety of microbial strains functions as a major defense mechanism to mitigate the damage of dissolved mercury [26,59,60].

In addition to the *mer* operon system, two other mechanisms were also responsible for Hg(II) reduction. First, *P. pantotrophus*, which does not carry *merA* genes (Fig. 6a), produced a significantly greater amount of O_2_^•–^ than the other two strains (Fig. 6c), likely due to the oxidative stress induced by mercury exposure [61,62]. This elevated level of O_2_^•–^ may lead to intracellular substantial mercury reduction in *P. pantotrophus*. Indeed, the addition of superoxide dismutase (SOD), an O_2_^•–^ scavenger, to the experimental matrices decreased Hg^0^ production by over 90% after a 9-h incubation (Fig. 6d), confirming the role of O_2_^•–^ in Hg^0^ formation. Second, Hg(II) reduction mediated by cytochrome *c*, a heme-containing protein, also contributed to the production of Hg^0^ by *A. ferrooxidans*. Production of Hg^0^ was significantly inhibited when KN_3_, a cytochrome *c* oxidase inhibitor, was added (Fig. 6e). PCR amplification further confirmed the presence of genes related to this pathway (i.e. *iro, coxB, coxC, coxA* and *rus*) in *A. ferrooxidans* (Fig. S6), and RT-qPCR revealed that the expression of these genes was apparently downregulated in the presence of KN_3_ (Fig. 6f). Since Fe(II) serves as a key electron donor for cytochrome *c*-mediated Hg(II) reduction [63], we conducted additional experiments with exogenous Fe(II) supplementation. The results showed that Fe(II) addition enhanced Hg^0^ production, and RT-qPCR analysis indicated upregulation of the corresponding cytochrome *c*-related genes, supporting the critical role of Fe(II) in facilitating Hg(II) reduction in this iron-oxidizing bacterium (Fig. 6e and f). Cytochrome *c* is an important electron transfer mediator [63], and it appears that for iron-oxidizing bacteria, cytochrome *c*-mediated mercury reduction may be an important mechanism complementing the *mer* operon-mediated reduction to mitigate mercury toxicity, particularly under iron-rich conditions. Regardless of the specific intracellular location where reduction occurs, the resultant elemental mercury is volatile and readily diffuses across the cellular membrane, ultimately escaping from the aqueous culture medium. However, we cannot rule out the possibility that reduction may also occur directly on the surface of intracellular nanoparticles. Further studies will be required to fully resolve the sequence of the dissolution and reduction events.

### Implications for metal biogeochemistry

**Figure 7.**
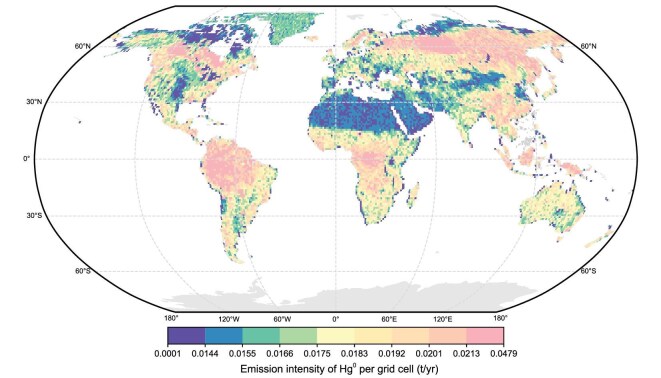
Global spatial distribution of the estimated annual Hg^0^ emission flux from soils driven by the reduction of HgS_NP_ in chemolithoautotrophic bacteria. The estimation is based on the following key constraints and assumptions: (i) the modeled spatial domain encompasses major terrestrial biomes (agricultural, desert, forest, grassland, wetland, tundra); and (ii) the flux is constrained by field-measured concentrations of HgS_NP_ and the relative abundance of chemolithoautotrophic bacteria capable of HgS reduction.

The observation that chemolithoautotrophic bacteria effectively convert Hg-bearing nanoparticles to form Hg^0^ unveils a previously unidentified, yet important source of Hg^0^ in natural microbial habitats. Chemolithoautotrophic microorganisms are prevalent in Hg-impacted regions such as terrestrial soils, marine sediments, deep-sea hydrothermal vents and acid mine drainage areas [64–67]. The three chemolithoautotrophic bacterial taxa (i.e. *Thiobacillus, Paracoccus* and *Acidithiobacillus*), to which the bacterial strains tested in this study belong, are widely distributed in terrestrial soils globally (Fig. S12). Moreover, nanoparticulate mercury represents an important fraction of the inorganic mercury pool in terrestrial soils (Table S2) [49,68–70]. Our modeling results show that the Hg^0^ emission due to the reduction of HgS_NP_ by these chemolithoautotrophic populations can reach 272.44 ± 134.99 tonnes per year (Fig. 7 and Fig. S20), with 50% and 90% confidence intervals of 179.38–362.75 and 40.58–536.87 tonnes per year, respectively. The uncertainty arises from two sources: uncertainty in the key parameters that control Hg^0^ production at each location, and the spatial heterogeneity across locations. Because the global estimate aggregates all locations, both sources are reflected in the final uncertainty range. (Figs S11–S19). This estimate integrates the reduction mechanism demonstrated in the laboratory with spatially resolved field data on the co-occurrence of HgS_NP_ and relevant chemolithoautotrophic bacteria across major terrestrial biomes. The flux from this newly discovered process is equivalent to that of geogenic Hg^0^ emissions (∼250 tonnes per year) [16,71], and similar to the emissions from cement production (∼236 tonnes per year), the fourth largest anthropogenic Hg^0^ emission source [72,73]. The significant role of chemolithoautotrophic metabolism in Hg^0^ production calls for targeted monitoring of chemolithoautotrophic bacterial activities in mercury-laden environments when evaluating the Hg emission potentials and warrants its inclusion in global mercury budget assessments. The release of volatile Hg^0^ from this newly identified process represents a missing component in the global atmospheric Hg^0^ budget. Our model provides a first-order approximation of the Hg^0^ emission flux potentially generated by this newly identified pathway. By synthesizing data from a range of experimental and observational studies, we derive a globally consistent estimate of this flux. While the modeling framework incorporates the key processes confirmed by laboratory evidence, certain environmental dynamics—such as mercury speciation changes, microbial community interactions and geochemical variability—are represented in a simplified manner due to limited empirical information at the global scale. These factors may affect net Hg^0^ release in natural settings. Continued empirical and modeling efforts will further refine these estimates and more precisely delineate the role of nanomineral-fueled chemolithoautotrophy in global mercury cycling.

The conventional wisdom on the origin of life dictates that minerals bearing crust-abundant and non-toxic elements, such as pyrite, serve as the primary energy sources to support life [74–76]. One important observation of this study is that not only are chemolithoautotrophic microorganisms capable of harnessing energy from HgS, a mineral containing toxic metals, but also that this process becomes sustainable and thus the dominant pathway of energy production when the minerals are bioavailable at the nanoscale. This highlights the importance of particle size, in addition to elemental composition, in re-defining the mineral–metal–microbe interactions that are critical to the field of metal biogeochemistry. Our discovery also remarkably expands the spectrum of mineral-supported chemolithoautotrophy and the understanding on how microbial life may evolve to cater for the diversity of energy production.

## MATERIALS AND METHODS

Detailed descriptions of materials and methods are presented in the Supplementary data.

## Supplementary Material

nwaf581_Supplemental_Files

## Data Availability

The input data, codes and modeling outputs used in this study are available on Zenodo: https://zenodo.org/records/17679469.
