# Supplementary material for: Nanomineral-fueled chemolithoautotrophy leads to substantial mercury emission
Source: Natl Sci Rev. 2025 Dec 19;13(5):nwaf581. doi: 10.1093/nsr/nwaf581 (PMC12949521; doi:10.1093/nsr/nwaf581)
Supplement: nwaf581_Supplemental_Files [file nwaf581_supplemental_files.zip › Supplementary materials-Chen et al.docx]

**SUPPLEMENTARY FILE**

Nanomineral-fuelled chemolithoautotrophy leads to substantial mercury emission

Zeyou Chen^1#^, Chenyang Zhang^1#^, Zhanhua Zhang^2#^, Qing Chang^1^, Cheng Gao^1^, Xiao Liang^1^, Xin Nie^1^, Long Chen^3^, Zhi Cao^1^, Yan Lin^1^, Pedro J. J. Alvarez^4^, Wei Chen^1^, Tong Zhang^1*^, Cong-Qiang Liu^5^

^1^ College of Environmental Science and Engineering, Ministry of Education Key Laboratory of Pollution Processes and Environmental Criteria, Tianjin Key Laboratory of Environmental Remediation and Pollution Control, Nankai University, Tianjin 300350, China.

^2^ Agro-Environmental Protection Institute, Ministry of Agriculture and Rural Affairs, Tianjin 300191, China.

^3^ School of Geographic Sciences, East China Normal University, Shanghai 200241, China.

^4^ Department of Civil and Environmental Engineering, Rice University, 6100 Main Street, Houston, TX 77005, USA.

^5^ School of Earth System Science, Tianjin University, Tianjin 300072, China.

^*^ Corresponding author: Tong Zhang (E-mail: zhangtong@nankai.edu.cn)

^#^ These authors contributed equally to this work.

**MATERIALS AND** **METHODS**

**1. Synthesis and characterization of nanoparticulate HgS**

Nanoparticulate HgS was synthesized using a literature method[1]. The experimental media were prepared with 0.1 M NaNO_3_ as the background electrolyte and 5 mM 4-(2-hydroxyethyl)-1-piperazineethanesulfonate (HEPES) to stabilize the pH at 6.8~7.0. Prior to use, the medium was deoxygenated by nitrogen gas purging and stored under anaerobic conditions in a glove box (Type A, Coy Laboratory Products Inc., USA). The Suwannee River humic acid (SRHA), Hg(NO_3_)_2_, and NaS solutions were introduced to establish predefined concentrations of 400 μM Hg(II), 400 μM S(II), and 40 mg C L⁻^1^ SRHA. The matrices were then kept static in the dark at room temperature inside the glove box.

**2. Characterization of nanoparticulate HgS and** **bulk HgS**

The morphology, particle size, and elemental composition of HgS_NP_ were characterized using transmission electron microscopy (TEM) and energy-dispersive X-ray spectroscopy (EDX). Nano Measurer software (v1.2.0) was used to analyze the geometric diameters of 100 particles observed in TEM images for statistical analysis. High-resolution TEM (HR-TEM) was employed to observe the lattice spacing of nanoparticles, which was used to verify the crystal phase determined by X-ray diffraction (XRD). The crystal structure of HgS_NP_ was analyzed using XRD with Cu Kα radiation (λ = 1.5418 Å). Commercial bulk HgS (Aladdin Inc., China) minerals were cleaned with dilute HNO_3_, rinsed with ultrapure water, freeze-dried, and then ground into powder using an agate mortar. The morphology and elemental composition of the bulk HgS minerals were characterized using a scanning electron microscope (SEM, JSM-7800F, JEOL, Japan) equipped with EDX. The specific surface area (BET) of the HgS minerals was measured using a multi-station surface area and porosity analyzer (ASAP 2460, Micromeritics, USA).

**3. Culturing of chemolithoautotrophic bacteria**

The sulfur-oxidizing bacteria selected for this study were *Thiobacillus thioparus* (ATCC 8158) and *Paracoccus pantotrophus* (ATCC 35512), obtained from the American Type Culture Collection (ATCC) and Beijing Beina Chuanglian Biotechnology Research Institute, respectively. The iron-oxidizing bacterium chosen was *Acidithiobacillus ferrooxidans* (ATCC 23270), also acquired from ATCC. Each bacterial strain was inoculated into its respective fresh culture medium at a 2% inoculation ratio. *T. thioparus* was incubated statically at 30 ℃ in a thermostatic biochemical incubator (SPL-250, Labotery, China), while *P. pantotrophus* and *A. ferrooxidans* were cultured in a full-temperature shaking incubator (HYG-A, Onuo Instruments, China) at 160 rpm and 30 ℃. Samples were taken every 12 hours post-inoculation, and the bacteria were stained with SYBR Green I dye (Thermo Fisher, USA). Bacterial counts in the cultures were then determined using a flow cytometer (Accuri C6 Plus, BD Biosciences, USA), and growth curves were plotted to monitor bacterial growth.

**4. Microbial mercury transformation assays**

The bacterial cultures were collected during the mid- to late-log phase and centrifuged at 4,000 rpm for 8 minutes. The supernatant was discarded, and the cell pellets were washed twice with phosphate-buffered saline (PBS). The centrifuged cells were then resuspended in culture media lacking Na_2_S_2_O_3_, CH_3_COONa, and FeSO_4_. The bacterial suspensions were transferred to fresh media at a 2% inoculation ratio, and 50 µmol L^–1^ HgS_NP_ and HgS mineral suspensions were added to the culture system. Each flask contained a total volume of 50 mL. The cultures were gently mixed and incubated at 30 ℃ in a static incubator. Bacterial counts in the cultures were then determined using a flow cytometer (Accuri C6 Plus, BD Biosciences, USA), and growth curves were plotted to monitor bacterial growth. Control groups were also established: (1) clean culture medium with an equivalent concentration of Hg(NO_3_)_2_ solution but without bacterial inoculation; (2) clean culture medium inoculated with the same ratio of bacteria but without any mercury addition. All other conditions were identical to those of the experimental groups.

To further investigate whether *A. ferrooxidans* employs additional pathways beyond the *merA*-mediated mercury reduction process, two experimental were established. After adding 0.25 µmol L^–1^ Hg(NO_3_)_2_ and 1 mM FeSO_4_ to each group, 5 mM KN_3_ (a cytochrome c oxidase inhibitor) were added to other group. The bacterial suspensions were transferred to the systems at a 2% inoculation ratio.

To observe the presence of nanoparticulate HgS within cells, a portion of the bacterial cultures was centrifuged, and the cell pellets were prepared for ultrathin sectioning for TEM analysis. The cells were washed twice with PBS, resuspended in pre-cooled 2.5% (v/v) glutaraldehyde fixative (G-CLONE, China), and then fixed with 1% (w/v) osmium tetroxide solution for 1 hour. The samples were sequentially dehydrated with ethanol and propylene oxide, embedded in Epon812 epoxy resin, and cured for 24 hours. The samples were sectioned into 60-90 nm ultrathin sections using a microtome. These sections were mounted on 200-mesh ultrathin carbon films and observed using TEM, with EDX analysis performed to determine the elemental composition of mercury-bound particles within the cells.

**5. Cellular uptake of nanoparticulate HgS**

To determine whether the internalization of HgS_NP_ into microbial cells requires ATP consumption, proton uncoupler carbonyl cyanide 3-chlorophenylhydrazone (CCCP) and ATP synthase inhibitors N,N′-dicyclohexylcarbodiimide (DCCD) and oligomycin were used to inhibit intracellular ATP production, and the concentration of intracellular mercury (Hg_int_) was measured. Bacteria were transferred to fresh media at a 2% inoculation ratio, and 5 μM CCCP, 10 μM DCCD, and 10 μM oligomycin solutions were added to the respective culture systems. The cultures were gently mixed and pre-incubated statically at 30 ℃ for 1 hour in a thermostatic incubator. Subsequently, equal concentrations of HgS_NP_ or Hg(NO_3_)_2_ solutions were added to the culture systems, with a total volume of 50 mL per flask. At various time points, samples from different treatment groups were collected using the sacrifice method to measure total protein, ATP concentration, and Hg_int_ levels in the cell lysates. ATP concentrations in the cell lysates were determined using an ATP assay kit (S0026, Beyotime, China), following the manufacturer’s instructions.

**6. Quantification of Hg^0^ and intracellular mercury**

To quantitatively analyze the production of Hg^0^, a bubbler setup was employed. The inlet of the sand core rod in the reaction flask was directly immersed in the culture solution. At different incubation time points, samples were taken using the sacrifice method. N_2_ gas was used as the carrier gas at a flow rate of 25 mL min⁻^1^, with continuous aeration for 10 minutes. The Hg^0^ was directly bubbled into a receiver bottle containing a digestion solution (HNO_3_: HCl: H_2_O = 5: 2: 9), where Hg^0^ was oxidized to Hg(II). At various time points, the culture medium from which Hg^0^ had been stripped was sampled in triplicate. In the first sample, 2% HNO₃ was added to measure the total mercury content, denoted as Hg_T_. The total mercury content in the system should be the sum of Hg_T_ and Hg^0^. The second sample was filtered through a 0.22-µm polyethersulfone membrane to remove the cells, and the filtrate was treated with 2% HNO_3_ to measure the mercury content, denoted as Hg_med_. The filtrate was then centrifuged at 14,000 rpm for 40 minutes, and the supernatant was treated with 2% HNO_3_ to measure the mercury concentration, denoted as Hg_sol_. The third sample was treated with 50 mmol L⁻^1^ 2,3-dimercapto-1-propanesulfonic acid (DMPS), mixed thoroughly, and incubated at 40 ℃ for 4 hours to desorb mercury adsorbed on the cell surface. After centrifugation at 14,000 rpm for 40 minutes, the supernatant was treated with 2% HNO_3_ to measure the mercury content, with the difference from Hg_sol_ denoted as Hg_ads_. The total intracellular mercury content (Hg_int_) was calculated as Hg_T_ – Hg_med_ – Hg_ads_. The total mercury concentration in each preserved sample was determined using cold vapor atomic fluorescence spectroscopy (CVAFS, Tekran 2600, Tekran, USA), following EPA Method 1631[2]. Control groups were also established using sterile culture medium containing the same concentration of nano-HgS but without bacterial inoculation. All other conditions were identical to those of the experimental groups.

The sample preparation method for determining different forms of intracellular mercury was adapted from Guo *et al.* (2023)[3]. First, the centrifuged cell pellets were washed twice with PBS solution and then added to 2 mL of 50 mmol L⁻^1^ DMPS solution. The cells were incubated at 40 ℃ for 4 hours to fully complex and desorb the surface-bound HgS_NP_ and Hg(II), followed by centrifugation at 5,000 rpm for 5 minutes. The cell pellets were then resuspended in PBS solution, and 50 µL of 20 mg mL⁻^1^ proteinase K was added, followed by cell lysis at 30 ℃ for 1 hour. After filtering the lysate through a 0.22-µm polyethersulfone membrane, the lysate was used for quantitative analysis of different intracellular mercury species. The lysate was analyzed using size exclusion chromatography (SEC) coupled with inductively coupled plasma mass spectrometry (ICP-MS) to quantify particulate and dissolved mercury. High-performance liquid chromatography (Alliance e2695, Waters, USA) equipped with a size exclusion column (Unitary NH2, Huapuke Instruments, China) was used for particle separation, with 1 mmol L⁻^1^ cysteine solution as the mobile phase. The effluent was then introduced into the ICP-MS (NexION 2000, PerkinElmer, USA) to detect the mercury signal peaks. The detailed QA/QC information on total Hg, Hg(II) and HgS_NP_ analyses is summarized in Table S3.

**7. Analysis of membrane integrity**

Cultures of *T. thioparus*, *P. pantotrophus*, and *A. ferrooxidans* were centrifuged and resuspended in their respective growth media. The bacterial suspensions were then incubated with either 50 µM Hg(NO_3_)_2_ or ATP production inhibitors (5 µM CCCP, 10 µM DCCD, and 10 µM oligomycin) at 30 °C. After incubation, cells were harvested by centrifugation, resuspended in 0.01 M PBS (pH 7.4), and stained with propidium iodide (PI, Thermo Scientific, P1304MP) at a final concentration of 10 µM. Following a 20 min incubation in the dark, fluorescence was measured using a microplate reader (Infinite M200, Tecan) with an excitation wavelength of 535 nm and an emission wavelength of 615 nm. Fluorescence intensity reflects the permeability of bacterial inner membranes under different treatments, providing an assessment of potential membrane damage or cytotoxicity. All assays were performed in triplicate.

**8. Analysis of sulfur oxidation products**

Cells were disrupted using an ultrasonic cell disruptor (JY92-IIN, Xinzhi, China) with an ice bath for 3 minutes. After sonication, the cell lysates were filtered through a 0.22-µm polyethersulfone membrane to remove intact cells, and the filtrate was used for the quantitative analysis of various sulfur anions. The cell lysates were stored at –20 ℃ until analysis. The samples were treated with an LC-Na column to remove heavy metals, and an ion chromatography system (ICS5000, Thermo Electron Corporation, USA) equipped with a KOH eluent was used to separate and quantify sulfur anions (S_2_O_3_^2^⁻, SO_3_^2^⁻, SO_4_^2^⁻) at different reaction time points. A gradient elution method was employed: starting with 20 mmol L⁻^1^ for 10 minutes, increasing to 60 mmol L⁻^1^ over the next 8 minutes, then decreasing back to 20 mmol L⁻^1^ for the final 2 minutes, with a flow rate of 1.1 mL min⁻^1^.

Elemental sulfur in the cultures was separated and quantified using high-performance liquid chromatography (HPLC). Methanol was added to the samples at a 5: 1 ratio (v: v) and extracted for 12 hours. After extraction, the cell lysates were filtered through a 0.22-µM polyethersulfone organic membrane, and the filtrate was used for the quantitative analysis of elemental sulfur. The specific parameters for the HPLC method were as follows: HPLC system (Alliance e2695, Waters, USA); column: Eclipse XDB-C18, 4.6 × 150 mm (Agilent); detector: VWD detector; detection wavelength: 265 nm; mobile phase: 100% methanol; flow rate: 1 mL min⁻^1^; column temperature: 30 ℃.

**9. Quantification of gene expression**

Genomic DNA was extracted from bacterial cells in the mid- to late-log growth phase using a bacterial genomic DNA extraction kit (DP302, Tiangen, China). The amplification system for *merA* and sulfur oxidation-related genes was then prepared, and target genes were amplified using a polymerase chain reaction (PCR) thermal cycler. The PCR products were subjected to electrophoresis at 130 V for 30 minutes in a horizontal nucleic acid electrophoresis system, and the presence of target gene bands was observed using a gel imaging system (MINI Space, Tanon, China).

The expression levels of the mercury reductase-encoding gene *merA* and sulfur oxidation-related genes were validated using RT-qPCR. Bacterial cultures were sampled at different time points, and cells were collected by centrifugation at 5,000 rpm for 5 minutes. The cell pellets were rapidly frozen with liquid nitrogen and stored at –80 ℃. Total RNA was extracted from these samples using Trizol reagent, and cDNA was synthesized using the FastKing One-Step gDNA Removal and cDNA Synthesis Premix Kit (KR118-02, Tiangen, China). The amplification experiments were performed on a fluorescence quantitative PCR instrument equipped with the QuantStudio TM6 system (Life Technologies, Thermo Fisher Scientific Inc.). The mRNA expression levels were calculated using the 2^–ΔΔCt^ method and normalized to the 16S rRNA gene. The primers for PCR were designed and synthesized by Genewiz Inc., China.

**10. Quantification of superoxide anion**

Bacterial cultures were centrifuged at 4,500 rpm for 9 minutes, and the supernatant was transferred to a new centrifuge tube. A specific amount of 3,3'-[1-(phenylamino)-3,4-tetrazolium]-bis(4-methoxy-6-nitro) benzenesulfonic acid sodium salt (XTT) was added to the supernatant to reach a final concentration of 125 μM. The mixture was gently mixed and incubated statically at 37 ℃ for 2 hours. The absorbance at 450 nm was then measured using a microplate reader. As a non-biological control, an equivalent concentration of XTT was added to clean culture media containing only HgS_NP_ and HgS minerals. To assess the role of O_2_^•–^ in mercury reduction, the O_2_^•–^ content was also measured in control experiments where superoxide dismutase (SOD) was added to eliminate O_2_^•–^.

**11. Molecular dynamic simulations**

The simulations of the cross-membrane process of HgS_NP_ and Hg(II)-DOM complex through the lipid membrane were performed using Gromacs program suite[4]. The lipid bilayer was composed of POPE and POPG at a 3:1 ratio, based on previous MD studies of bacterial membranes[5]. The Slipids-2020 force field[6] was used to describe the POPE and POPG phospholipids together with Na^+^ cation. The water molecules were simulated using SPC/E water model parameters. The UFF forcefield[7] and Qeq charge model were used to describe the inorganic HgS nanocluster. Fulvic acid was chosen as a representative DOM fragment due to its prevalence, structural diversity (carboxyl, hydroxyl, aromatic groups), and known interaction with Hg(II). DOM fragments were DFT-optimized prior to MD simulations. The Hg(II)-DOM complex was optimized through density functional theory (DFT) calculation at PEB0 functional and def2-SVP basis set and then parameterized using GAFF force field and RESP charge model. All these topology files of these molecules were generated directly using the AuToFF web server. These simulations were carried out at a pressure of 1.01 × 10^5^ Pa using Berendsen's barostat[8] with an isothermal compressibility constant of 4.5 × 10^–5^. Velocity-rescale thermostat[9] with a relaxation constant of 1 ps was used to control the temperature at 298.15 K. Periodic boundary conditions were applied in all directions. Particle-mesh Ewald (PME) method[10] with a cut-off distance of 15 Å was applied to treat the electrostatic interactions and the van der Waals forces. All bonds involving hydrogen atoms were constrained with the LINCS algorithm. There are forty configurations for umbrella sampling generated by pulling the HgS_NP_ and Hg(II)-DOM complex through the membrane system along the Z-axis using steered molecular dynamics simulations. Each configuration was then performed an additional 1 ns equilibrium process at constant NPT ensemble with the same simulation parameters as above. After the system pressure had reached an equilibrium, 5 ns molecular dynamics sampling was performed for the next calculation of free energy profiles through the weighted histogram analysis method.

**12. Estimation of global Hg^0^ emissions by chemolithoautotrophic bacteria**

12.1. Model framework for global soil Hg^0^ emission estimation

We developed a spatially explicit modeling framework to quantify global soil Hg^0^ emissions mediated by the three chemolithoautotrophic bacteria genera (*Thiobacillus*, *Paracoccus*, and *Acidithiobacillus*). This framework mechanistically integrates the fundamental physiological constraints of mercury-transforming bacteria with the biogeochemical limitations imposed by bioavailable mercury substrates. Grounded in first-principles microbial ecology and mercury biogeochemistry, this model advances empirical approaches by coupling bottom-up biological processes with top-down geochemical constraints.

The theoretical foundation follows a mass-balance formulation, in which Hg^0^ emissions from each spatial unit are dynamically constrained by the lesser of two factors: (i) bacterial transformation capacity and (ii) geochemical availability of reducible mercury species. This dual-constraint mechanism ensures that emissions cannot exceed either biological potential or chemical substrate supply. The framework also resolves competition between biological reduction and chemical stabilization processes, yielding a more realistic representation of terrestrial mercury cycling.

12.1.1. Fundamental governing equations

The hierarchical model is mathematically expressed as the following coupled equations:

$\text{Flux}_{\text{total}}\text{ =}\sum_{\text{i}\text{=1}}^{\text{n}} \text{Flux}_{\text{i}}$ (E1)

$\text{Flux}_{\text{i}}\text{=}\text{min}$($\text{Flux}_{\text{bac}}\text{ }$, $\text{Flux}_{\text{Hg}}$) (E2)

$\text{Flux}_{\text{bac}}$ =$\text{SBA}_{\text{x}}\text{ ×}\text{PR}_{\text{Hg}^{\text{0}}}\text{ ×}\text{Area}_{\text{x}}$ (E3)

$\text{Flux}_{\text{Hg}}$ = $\text{T}_{\text{Hg}}\text{ × }\text{P}_{\text{HgS}_{\text{NP}}}$ (E4)

where *Flux*_total_ represents the cumulative Hg^0^ flux from all spatial grids; *n* is the different land use types; *Flux_i_* represents the Hg^0^ emission flux per 1° × 1°spatial grid; *Flux_bac_* denotes the Hg^0^ production potential constrained by the bacterial abundance in each grid cell; *Flux_Hg_* reflects the Hg^0^ generation capacity limited by the available HgS nanoparticles (HgS_NP_) in each grid cell. *SBA_x_* is the soil bacterial abundance per unit area (cells/m^2^). $\text{PR}_{\text{Hg}^{\text{0}}}$ is the experimentally determined conversion efficiency of HgS_NP_ to Hg^0^ by the three chemolithoautotrophic bacteria in this study (0.59 × 10^–12^ – 1.75 × 10^–12^ g·cell^-1^·h^-1^); *Area_x_* corresponds to the land use type-specific surface area within each grid cell (m^2^). *T_Hg_* is the soil total mercury concentration (μg·kg^-1^); $\text{P}_{\text{HgS}_{\text{NP}}}$ is determined by the relationship between global surface soil organic carbon (SOC) data and the measured proportion of HgS_NP_ to total mercury (THg) across varying SOC concentrations.

In this formulation, Hg^0^ fluxes from each grid cell are simultaneously constrained by bacterial transformation potential and available Hg substrate, with the effective emission flux given by the more limiting term. This ensures physical consistency across spatial units and avoids unrealistically high emission estimates.

12.1.2. Spatial discretization and habitat characterization

The global terrestrial surface was discretized into a 1° × 1° grid system (approximately 111 km × 111 km at the equator), providing an optimal balance between computational tractability and ecological representativeness. For each grid cell, fractional coverage of habitat types was derived from remote-sensing and ecological databases (<http://www.globallandcover.org>). We used GlobeLand30 (2010), a 30 m global land-cover product generated by the National Geomatics Center of China from Landsat TM/ETM+ and HJ-1 imagery using a pixel-object-knowledge classification approach. The dataset distinguishes ten primary land-cover types: cropland, forest, grassland, shrubland, wetland, water body, tundra, artificial surface, bare land, and permanent snow/ice. The overall accuracy of GlobeLand30 (2010) has been reported to be over 80% [11].

Soil organic-carbon and textural data (e.g., SOC content, clay fraction) were obtained from SoilGrids 2.0 (<https://soilgrids.org/>) at 250 m resolution, while total soil-Hg concentration data were sourced from our previous study [12]. Grid cells lacking valid habitat coverage were excluded to reduce computational load. All spatial datasets were harmonized through a standardized raster-processing pipeline comprising: (i) coordinate reference unification to WGS84, (ii) bilinear resampling for spatial alignment, (iii) outlier detection and quality control, and (iv) standardized imputation for missing values.

12.1.3. Spatial probabilistic modeling of bacterial abundance

Spatial probabilistic modeling of bacterial abundance was conducted using a Monte Carlo approach to propagate uncertainty from sample measurements to global grids. We divided the global soil into six major habitats: agriculture, desert, forest, grass, wetland, tundra (Figure S11). Habitat-specific bacterial density distributions were derived from metagenomic datasets spanning diverse terrestrial biomes (Figures S12-13). Distributional forms (normal vs. log-normal) were selected based on statistical descriptors of skewness and kurtosis and verified using Anderson-Darling goodness-of-fit tests. This ensured faithful representation of biological variability. A Monte Carlo protocol with 500 iterations per grid cell was implemented to propagate abundance uncertainty through all downstream computations. This number of iterations ensures a stable convergence of statistical moments (mean and variance) while maintaining computational efficiency at the global scale. In the Monte Carlo simulations, the parameters *SBA_x_* (soil bacterial abundance per unit area) was explicitly constrained to avoid non-physiological zero values. This constraint was implemented because our metagenomic analyses empirically demonstrated that all the three chemolithoautotrophic bacterial genera, albeit at low abundances, were consistently detectable (*SBA_x_* > 0).

12.1.4. SOC-dependent modeling of HgS_NP_ proportion

12.1.4.1 SOC interval classification scheme

We developed a probabilistic approach to characterize the relationship between SOC concentration (Figure S14) and the proportion of HgS_NP_ to THg. SOC concentrations were categorized into different levels, and the HgS_NP_ proportion for each interval was empirically determined from extensive field surveys (Figure S15).

Between August 2023 and November 2024, we sampled 92 representative paddy sites across China’s major rice-growing regions during the drainage period—conditions comparable to the crop-growth season. Soils (0-20 cm) were collected after removing visible plant residues, sealed in Ziploc bags, kept on ice, and transported immediately to the laboratory.

Hg-NPs were extracted following established protocols. Briefly, soils were dispersed in 10 mM tetrasodium pyrophosphate (TSPP; 1:10 w/v), sonicated for 15 min (40 kHz), shaken for 24 h, and centrifuged at 8,800 × g for 20 min to impose a nominal 100 nm cut-off. Pellets were twice re-extracted with ultrapure water. The extracts were determined after oxidation with 2% (v/v) BrCl and neutralization with NH_2_OH·HCl by atomic fluorescence spectrometry (LOD 0.10 μg L^-1^). Spike–recovery tests in paddy soils yielded 87.1 ± 6.5% (n = 3).

Crystallographic analysis of Hg-NPs extracted from paddy soils was performed using X-ray diffraction (XRD; Ultima IV, Rigaku, Japan) with Cu Kα radiation (λ = 1.5418 Å). The morphology and elemental composition of Hg-NPs were examined by transmission electron microscopy (TEM; JEM-2800, JEOL, Japan) coupled with energy-dispersive X-ray spectroscopy (EDS), selecting soils based on their THg concentrations. Prior to analysis, Hg-NP extracts were sonicated for 15 min, and 5 μL aliquots were deposited onto copper grids with ultrathin carbon films. Images were processed using Gatan Digital Micrograph Software.

12.1.4.2 Distribution fitting and selection protocol

For each SOC interval, several candidate probability distributions (Beta, Normal, Uniform) were fitted by maximum likelihood estimation. Because HgS_NP_ proportion data are bounded (0, 1), Beta distributions were generally preferred; Normal or Uniform distributions were applied when statistical constraints dictated, such as when the sample size was too small to reliably estimate Beta parameters or when the data exhibited near-constant values that hindered model convergence.

12.1.4.3 Spatial interpolation and assignment algorithm

Grid cells were classified into predefined SOC intervals based on their mean SOC values from SoilGrids. For each SOC interval, we fitted probability distributions to the observed HgS_NP_ proportion data using maximum likelihood estimation. During Monte Carlo simulations (n = 500 iterations per grid cell), SOC values were first sampled from normal distributions defined by each cell's mean and standard deviation. Based on the sampled SOC value, corresponding HgS_NP_ proportions were then drawn from the fitted interval-specific distributions. This approach propagated uncertainty from both SOC measurements and HgS_NP_ proportion variability, generating probabilistic estimates of HgS_NP_ distributions across global soils. In the Monte Carlo simulations, the parameter $\text{P}_{\text{HgS}_{\text{NP}}}$ (representing the proportion of HgS_NP_ to THg) was constrained to avoid non-physiological zero values. This constraint was implemented because HgS_NP_, as a component of the natural soil mercury pool, are ubiquitously present even at trace levels ($\text{P}_{\text{HgS}_{\text{NP}}}$ > 0). Consequently, the probability distribution for this parameter was truncated at a defined lower bound greater than zero to reflect this real-world condition.

12.1.5. Multi-stage uncertainty quantification framework

To rigorously propagate both aleatory and epistemic uncertainties through the modeling chain, we implemented a multi-stage Monte Carlo framework. This approach systematically quantifies error propagation from parameter estimation to final Hg^0^ flux prediction.

12.1.5.1 Bacterial abundance uncertainty propagation

Uncertainty in habitat-specific bacterial abundance was quantified using Monte Carlo sampling—500 realizations per grid cell—to efficiently explore the multivariate parameter space. Habitat-area-weighted spatial aggregation incorporated both abundance uncertainty and spatial heterogeneity. Probabilistic sampling employed parametric distribution fitting (normal or log-normal) based on observed abundance data characteristics. Distribution parameters were estimated from empirical data, with automatic selection between normal and log-normal distributions based on data skewness. Uncertainty propagation captured both sampling variability and habitat area effects through multiplicative scaling.

12.1.5.2 *Flux_bac_* uncertainty propagation

Uncertainty in bacterial transformation fluxes (*Flux_bac_*) was propagated using Monte Carlo simulation with 500 simulations per grid cell. Independent normal distributions were assumed for both spatially-explicit bacterial abundance and production rate, with *Flux_bac_* calculated as their multiplicative product. Spatial partitioning along longitude enabled parallel processing of large datasets through a divide-and-conquer approach. Uncertainty metrics including mean, standard deviation, coefficient of variation, and quantiles were derived empirically from the simulated flux distributions.

12.1.5.3 HgS_NP_ proportion uncertainty characterization

Uncertainty in mercury-substrate availability was described using a conditional-probability framework. 500 Monte Carlo simulations per grid cell ensured proportional representation of SOC regimes. For each simulation, a SOC value was first sampled from a normal distribution defined by the grid cell’s mean and standard deviation of SOC. Then, based on the sampled SOC value, the corresponding SOC interval was determined, and a HgS_NP_ proportion value was sampled from the distribution fitted to the HgS_NP_ proportion observations in that interval. This two-step sampling process captured the uncertainty in both SOC and the relationship between SOC and HgS_NP_ proportion.

12.1.6. Final emission constraint implementation

Integration of biological and geochemical constraints was achieved through a Monte Carlo simulation framework. Physical non-negativity was enforced by truncating sampled values at zero. The limiting-factor operator (*Flux_i_ = min(Flux_bac_, Flux_Hg_)*) was evaluated empirically through repeated sampling, with selection probabilities calculated as relative frequencies (Figures S19-20). Spatial patterns of constraint dominance were systematically quantified throughout the domain. This integration yields probabilistically constrained global Hg^0^ emission estimates that honor both microbial-kinetic potential and geochemical substrate limitations.

12.1.7. Additional modeling results

The spatial distributions of modeled Hg^0^ emission potentials—constrained separately by bacterial activity (*Flux_bac_*) and bioavailable Hg substrate (*Flux_Hg_*). These results reveal distinct biogeographic patterns that reflect the differential influence of microbial and geochemical factors across global terrestrial ecosystems. *Flux_bac_*, representing the Hg^0^ production potential limited by the abundance and activity of the three chemolithoautotrophic bacterial genera, exhibits pronounced heterogeneity correlated with soil bacterial biomass and habitat type (Figure S16). *Flux_Hg_*, which denotes the Hg^0^ generation capacity limited by the availability of HgS_NP_, closely tracks the spatial variability of soil total Hg content and the SOC-modulated proportion of HgS_NP_ (Figure S17). The model simulations yielded a global soil Hg^0^ emission estimate of 272.44±134.99 t·yr^-1^. The uncertainty of this estimate is quantified by a 50% confidence interval of (179.38, 362.75) t·yr^-1^ and a 95% confidence interval of (40.58, 536.87) t·yr^-1^.

12.2. Metagenomic sample quality control and taxonomic annotation

Soil metagenomes used in this study (Table S4) were obtained from the European Nucleotide Archive (ENA; https://ebi.ac.uk/ena/) and the NCBI Sequence Read Archive (SRA; https://ncbi.nlm.nih.gov/sra/). To minimize potential biases, the following selection criteria were applied:

(i) Only whole-genome shotgun (WGS) metagenomes generated using paired-end sequencing were included; datasets derived from PCR amplification or clone-based approaches were excluded.

(ii) Metagenomes obtained from laboratory-scale treatment systems, experimental field trials, or heavily contaminated sites were excluded to avoid confounding effects of anthropogenic disturbances.

(iii) Only datasets generated using Illumina shotgun sequencing platforms were retained; those produced with Roche 454 or ABI SOLiD technologies were removed.

(iv) Each dataset was required to include precise geographic coordinates and habitat metadata to enable robust spatial analysis.

(v) To prevent spatial redundancy, samples collected within 0.5° of each other (based on geographic distance) were filtered out.

Raw sequencing reads of selected metagenomes underwent stringent quality control using Fastp (v0.23.4) [13] to remove low-quality and technical artifacts. Reads shorter than 36 bp or with base quality scores below Q30 were discarded. The --trim_poly_g and --trim_poly_x options were enabled to eliminate homopolymeric G and other mononucleotide runs commonly introduced by sequencing errors. The resulting high-quality reads were assessed using FastQC (http://www.bioinformatics.babraham.ac.uk/projects/fastqc/), and the reports were summarized with MultiQC (v1.0) [14]. A subsequent manual screening step ensured that only metagenomes passing the Sequence Quality Histograms evaluation were retained. After quality control, a total of 165 metagenomes were selected for downstream analysis.

Taxonomic classification of the quality-filtered reads was performed using Kraken2 (v2.1.2) [15]with default parameters. Relative abundances were normalized to the total prokaryotic content of each sample to enable consistent cross-sample comparisons.

**
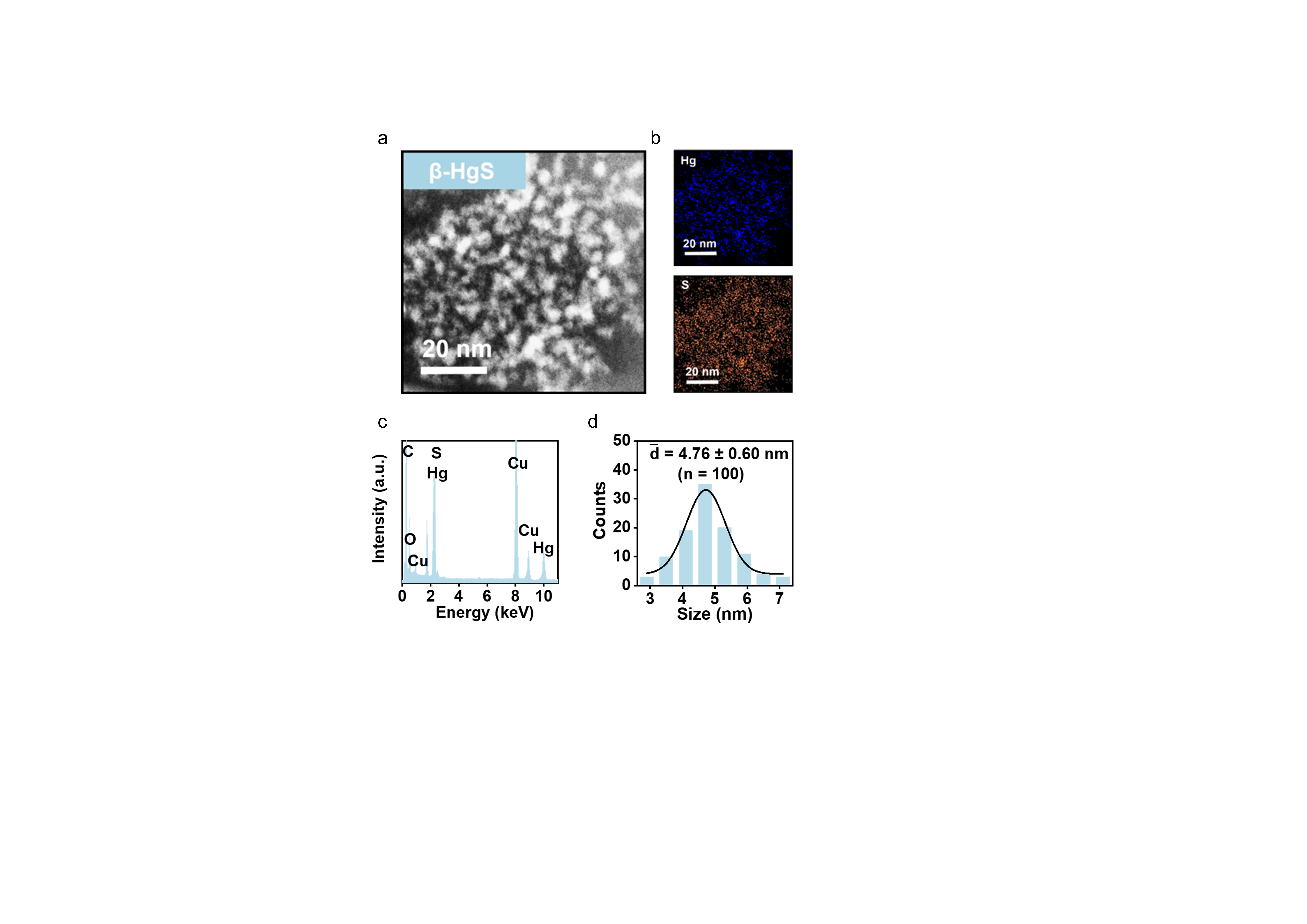
**

**Figure S1.** Transmission electron microscopy (TEM) image (a), energy dispersive X-ray spectroscopy (EDX) mapping (b) and spectrum (c), and particle size distribution (d) of the as-prepared HgS_NP_.


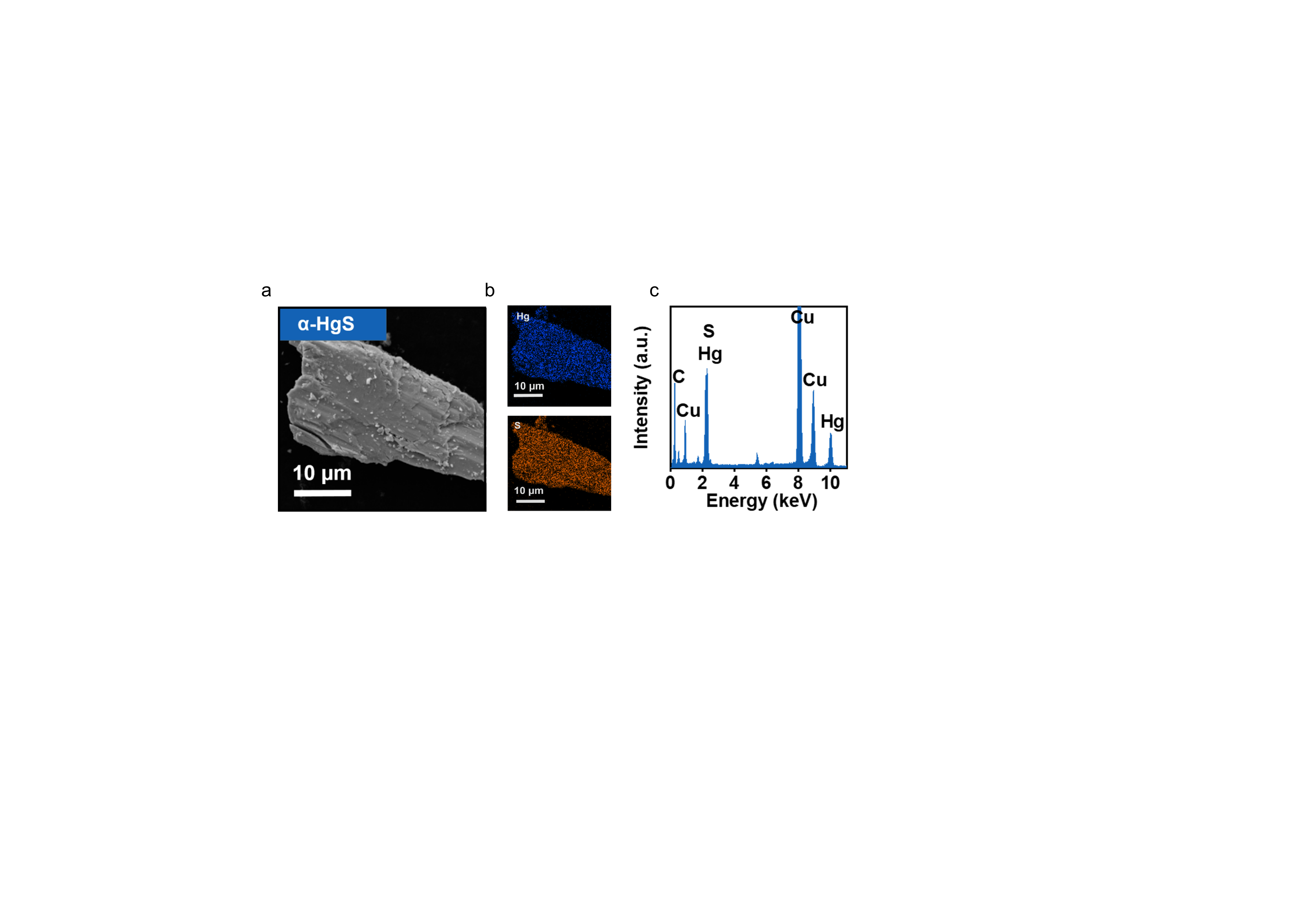


**Figure S2.** Scanning electron microscopy (SEM) image (a), EDX mapping (b) and spectrum (c) of the bulk HgS minerals.


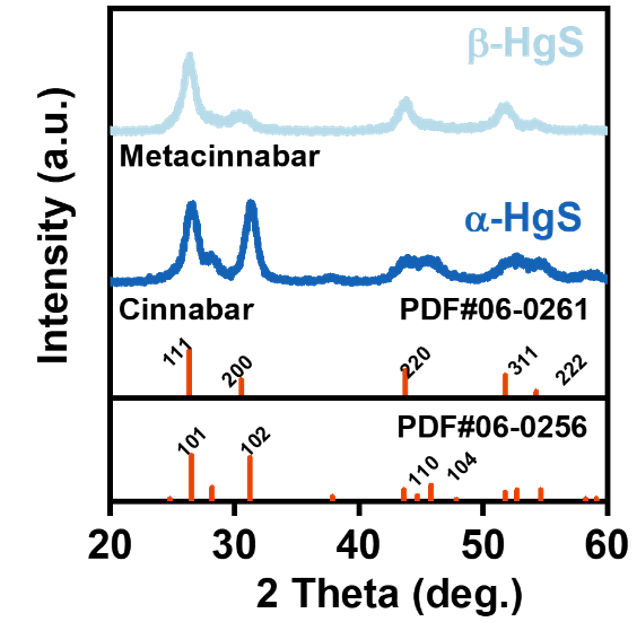


**Figure S3.** X-ray diffraction (XRD) patterns of as-prepared HgS_NP_ and bulk HgS minerals.


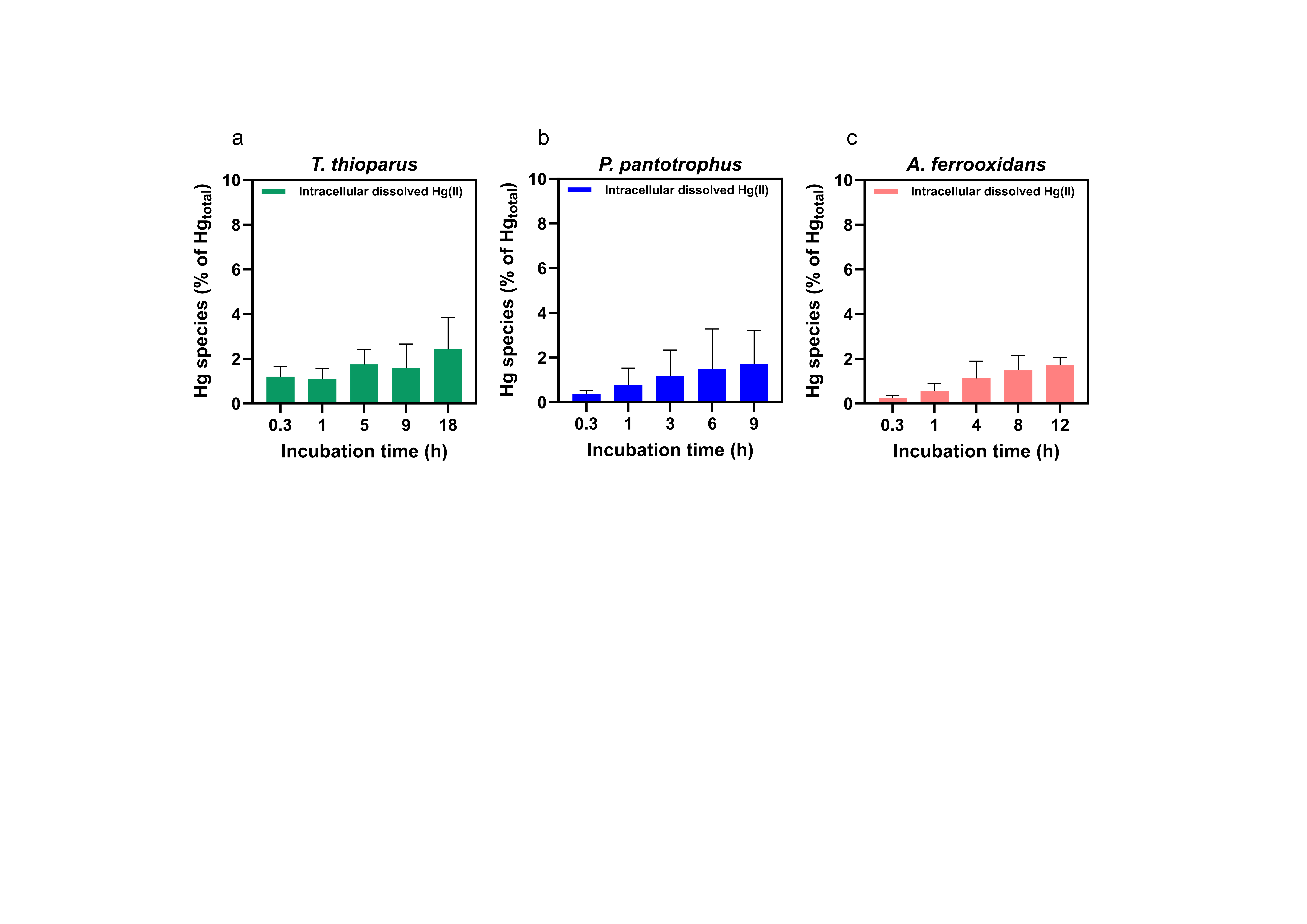


**Figure S4.** Changes in concentrations of Hg(II) in the cytoplasm of *T. thioparus* (a), *P. pantotrophus* (b) and *A. ferrooxidans* (c) strains after exposure to bulk HgS. Error bars represent ± 1 standard deviation of triplicates.


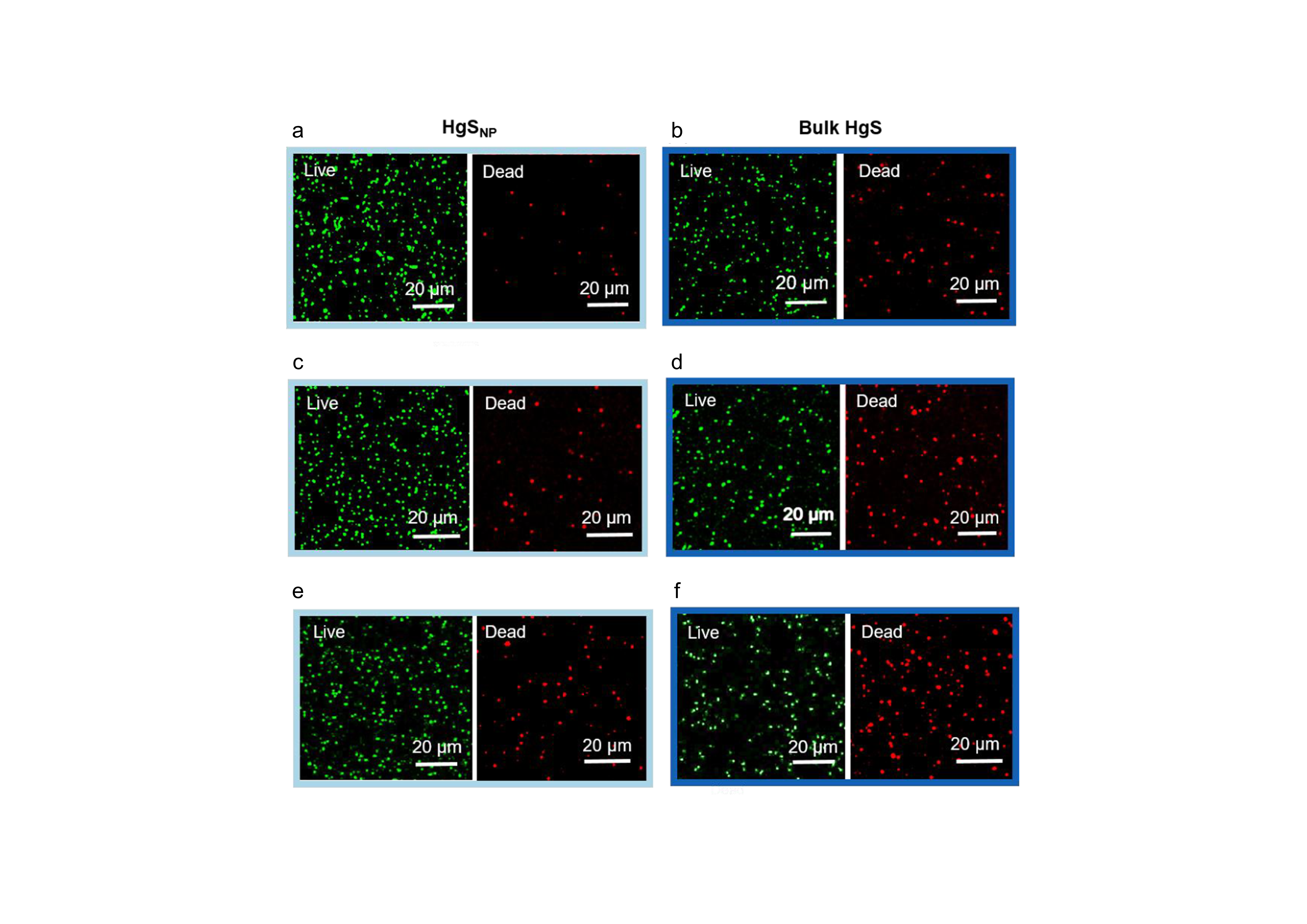


**Figure S5.** Confocal microscopy images showing *T. thioparus* (a, b), *P. pantotrophus* (c, d) and *A. ferrooxidans* (e, f) strains after exposure to HgS_NP_ (a, c, e) and bulk HgS (b, d, f) 260 h, live cells were stained in green by SYTO 9 while dead cells were stained in red by PI.


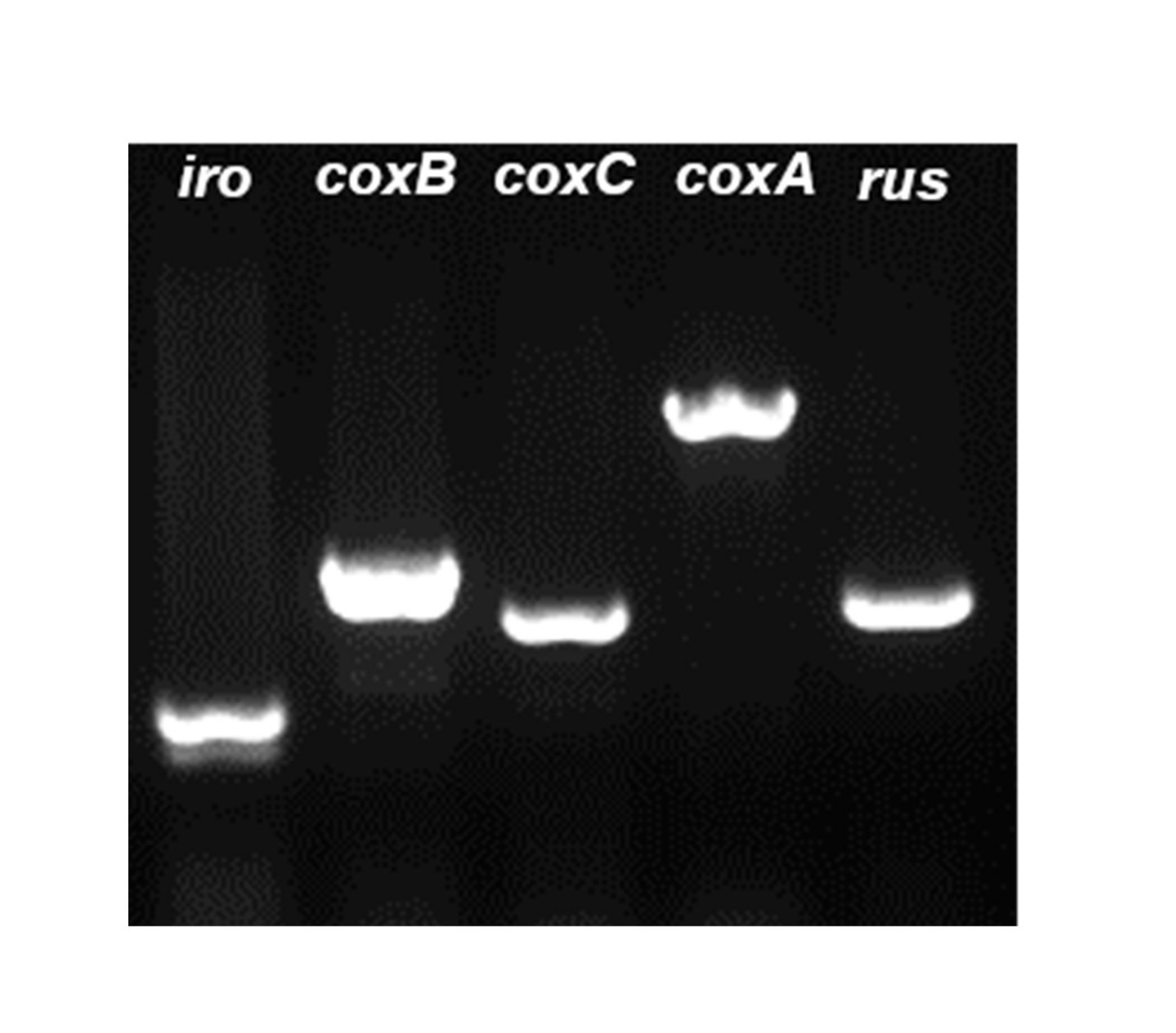


**Figure S6.** Agarose gel electrophoresis results of polymerase chain reaction (PCR) products from *A. ferrooxidans*. These genes encode iron oxidase, ceruloplasmin and cytochrome *c* oxidase in cells.

**
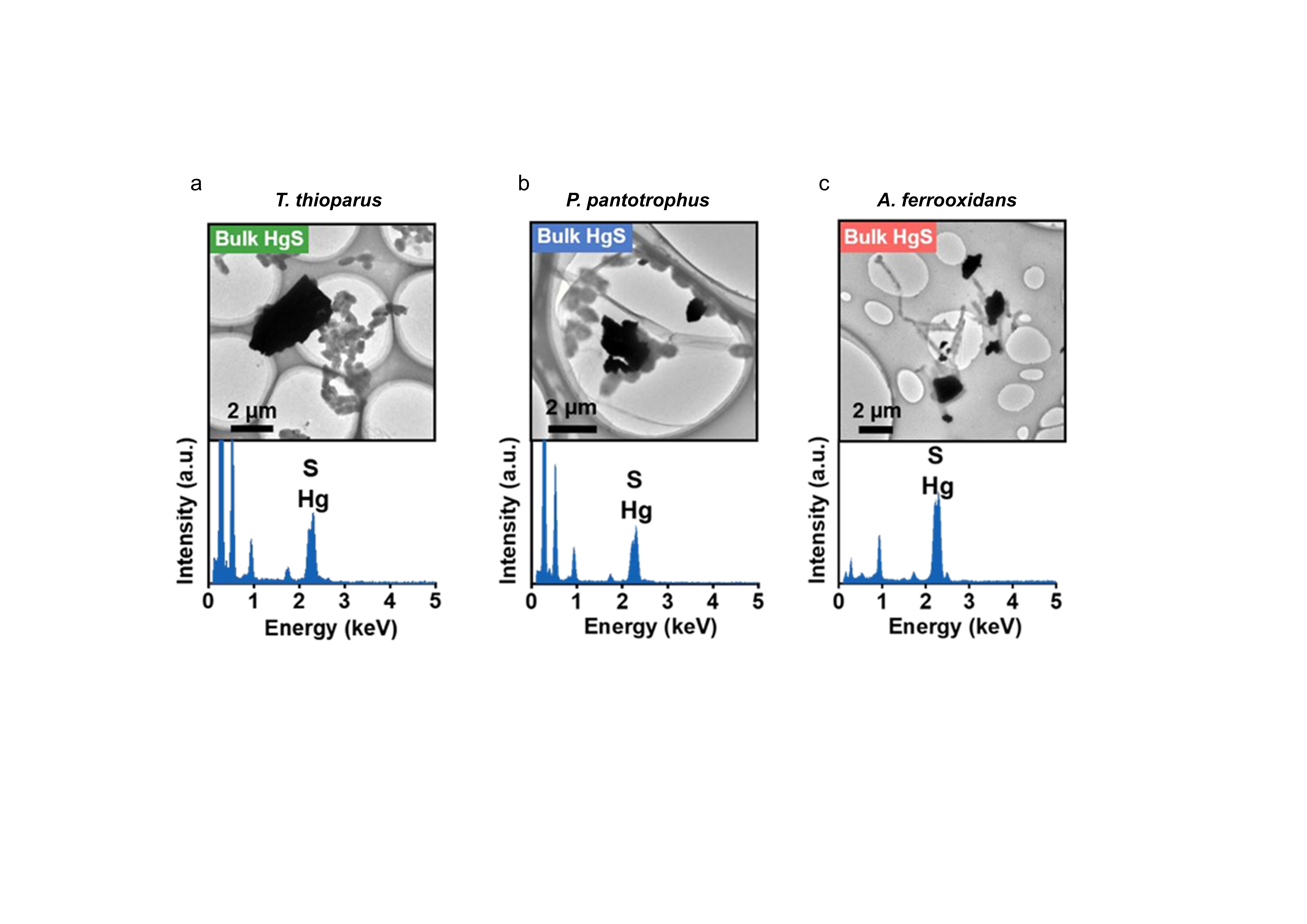
**

**Figure S7.** a–c, Transmission electron microscopy (TEM) images of thin section of *T. thioparus* (a), *P. pantotrophus* (b) and *A. ferrooxidans* (c) cells after 6-h exposure to bulk HgS, and energy-dispersive X-ray spectroscopy (EDX) spectra of bulk HgS.

**
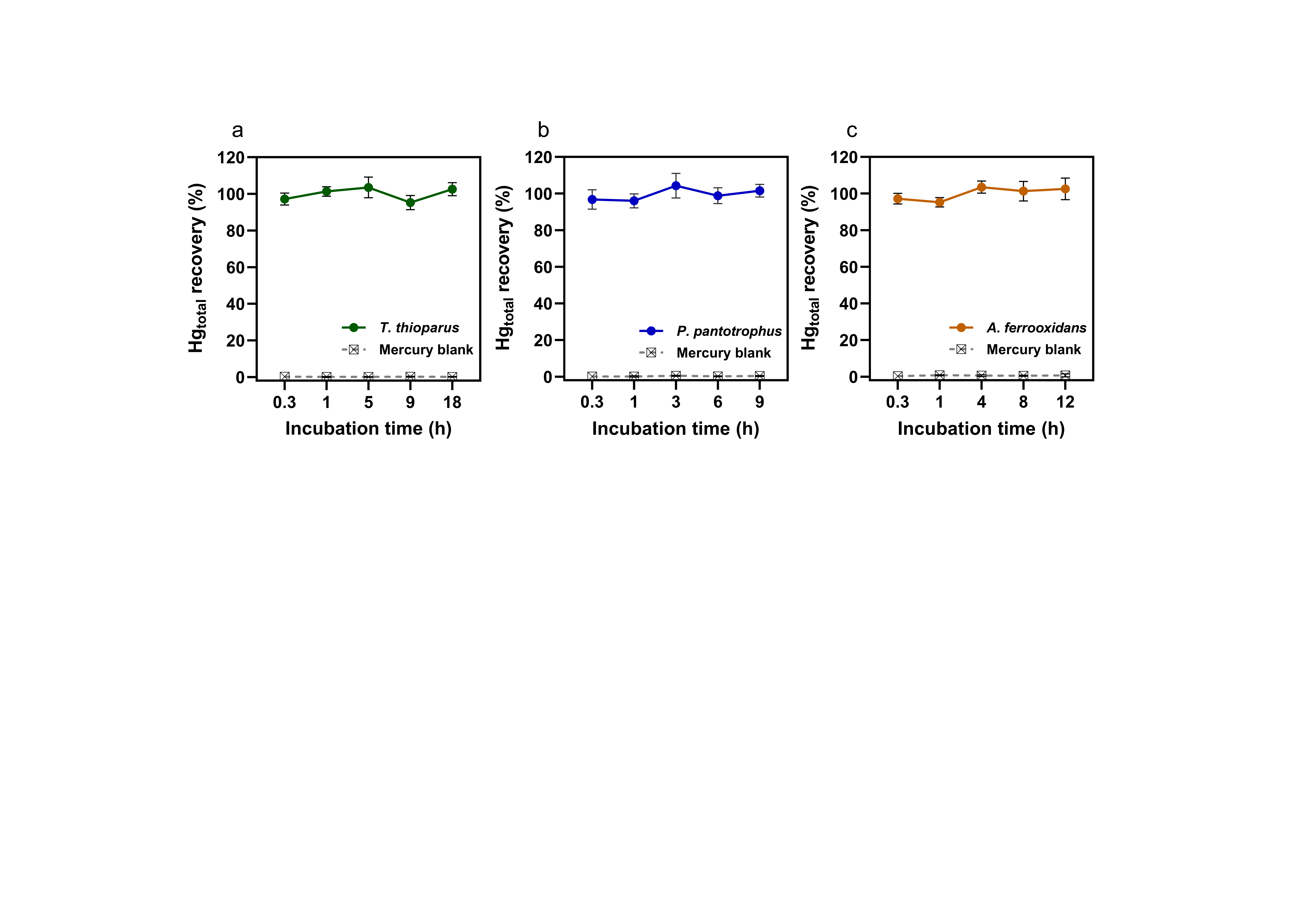
**

**Figure S8.** Total mercury recovery from *T. thioparus* (a), *P. pantotrophus* (b) and *A. ferrooxidans* (c) upon exposure to 50 μM HgS_NP_. Mercury blank represents active cultures without mercury addition. Error bars represent ± 1 standard deviation of triplicates.


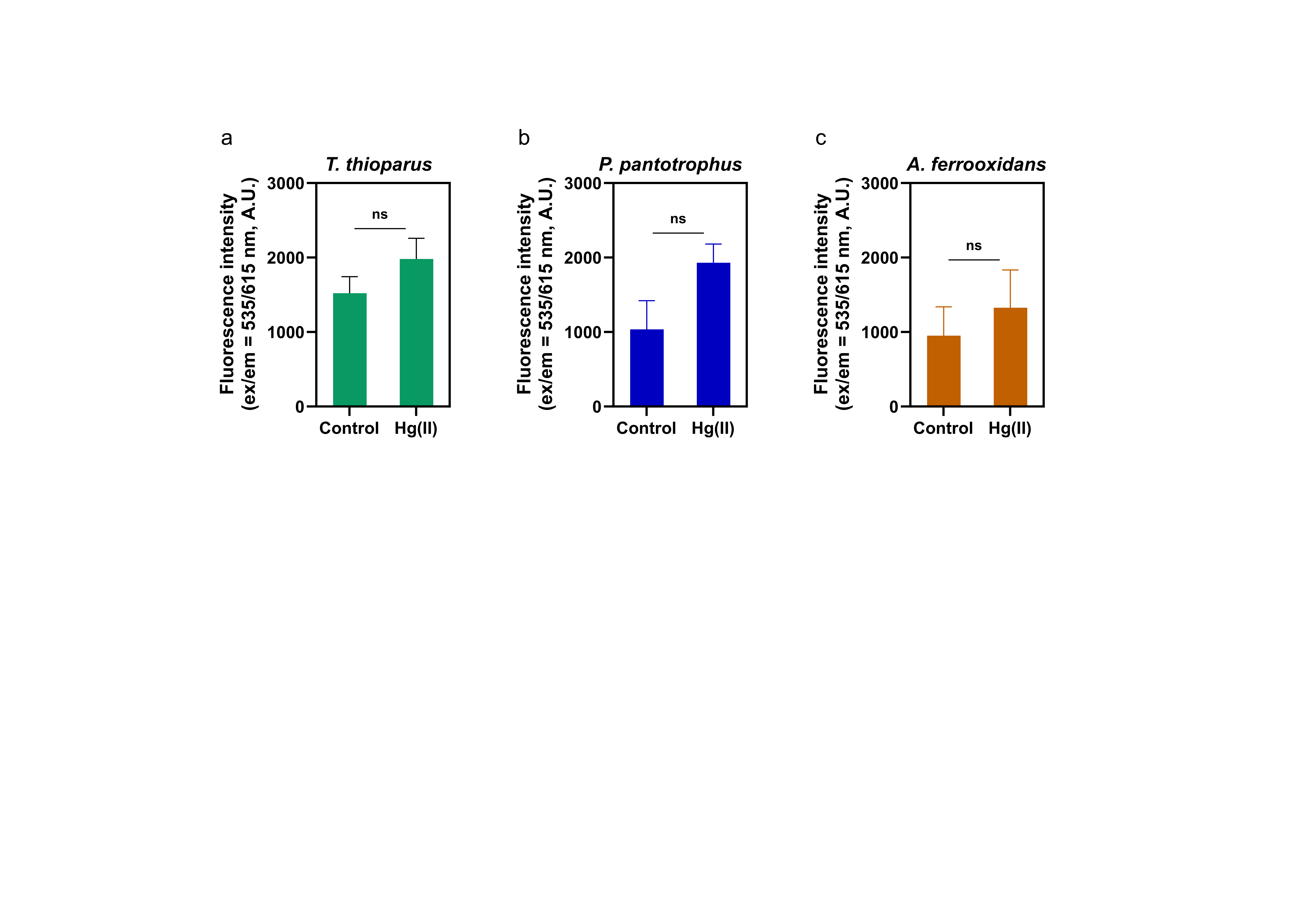


**Figure S9.** Mortality assessed by loss of membrane integrity following treatment with 50 µM Hg(II). Propidium iodide is a membrane-impermeant dye that fluoresces upon binding to nucleic acids in cells with compromised membranes. Error bars represent ± 1 standard deviation of triplicates.

**
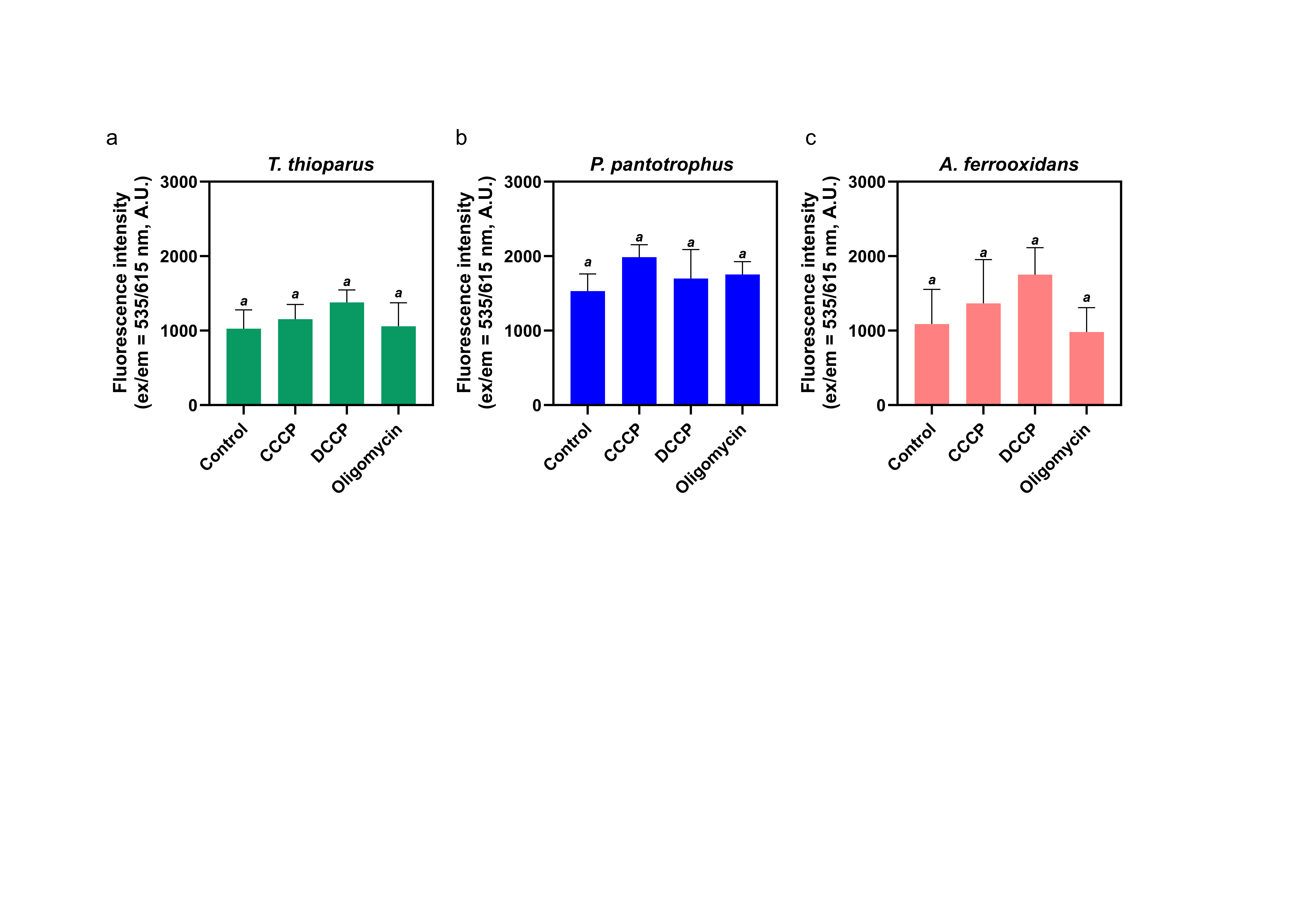
**

**Figure S10.** Mortality assessed by loss of membrane integrity following treatment with ATP inhibition experiments (CCCP, DCCD, oligomycin). Propidium iodide is a membrane-impermeant dye that fluoresces upon binding to nucleic acids in cells with compromised membranes. Error bars represent ± 1 standard deviation of triplicates.


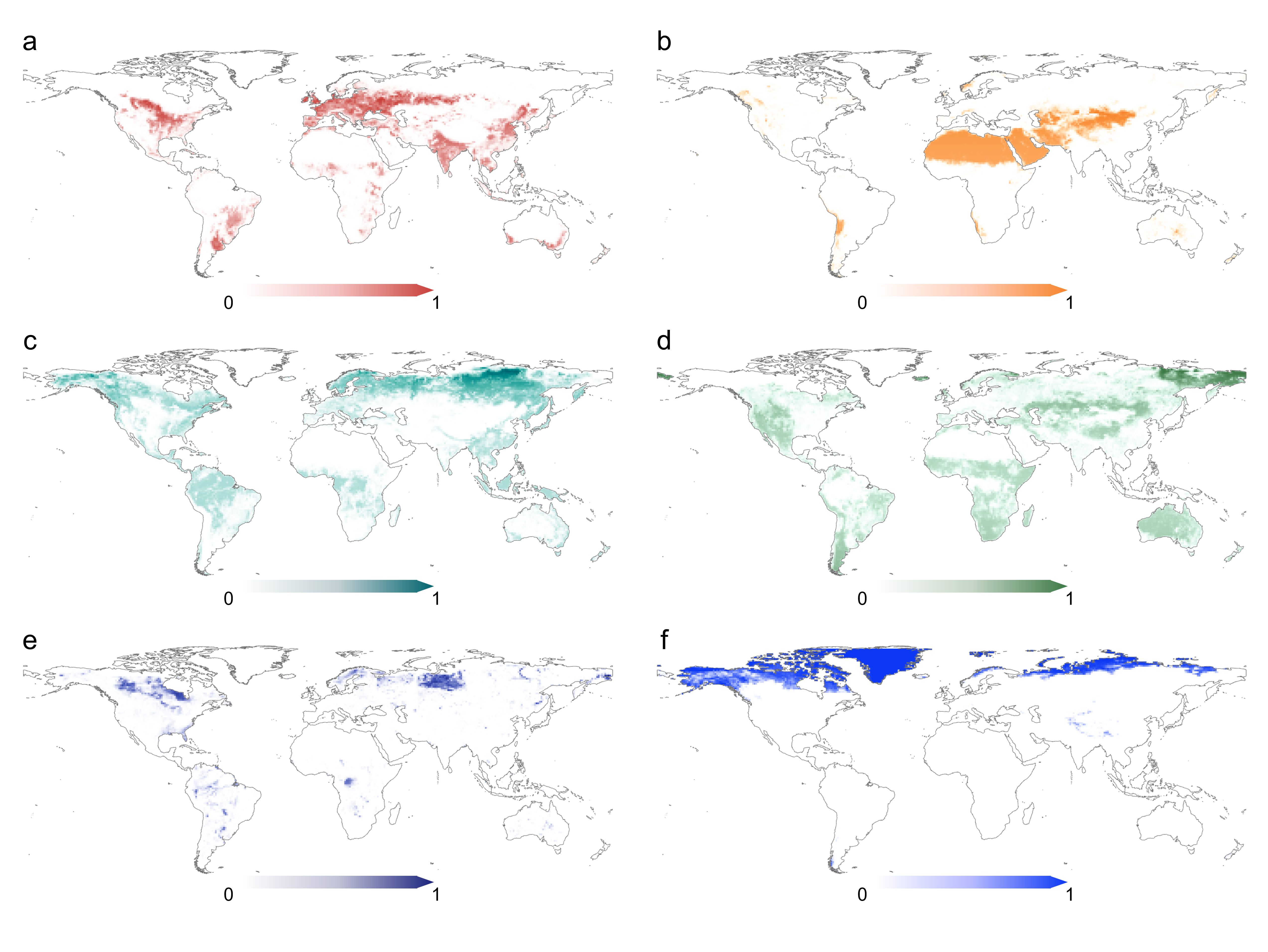


**Figure S11.** Proportions of agricultural, forest, grassland, wetland, tundra, desert, and other habitat categories within each 1° × 1° terrestrial grid cell. Data derived from GlobeLand30 (2010) global land-cover classification.


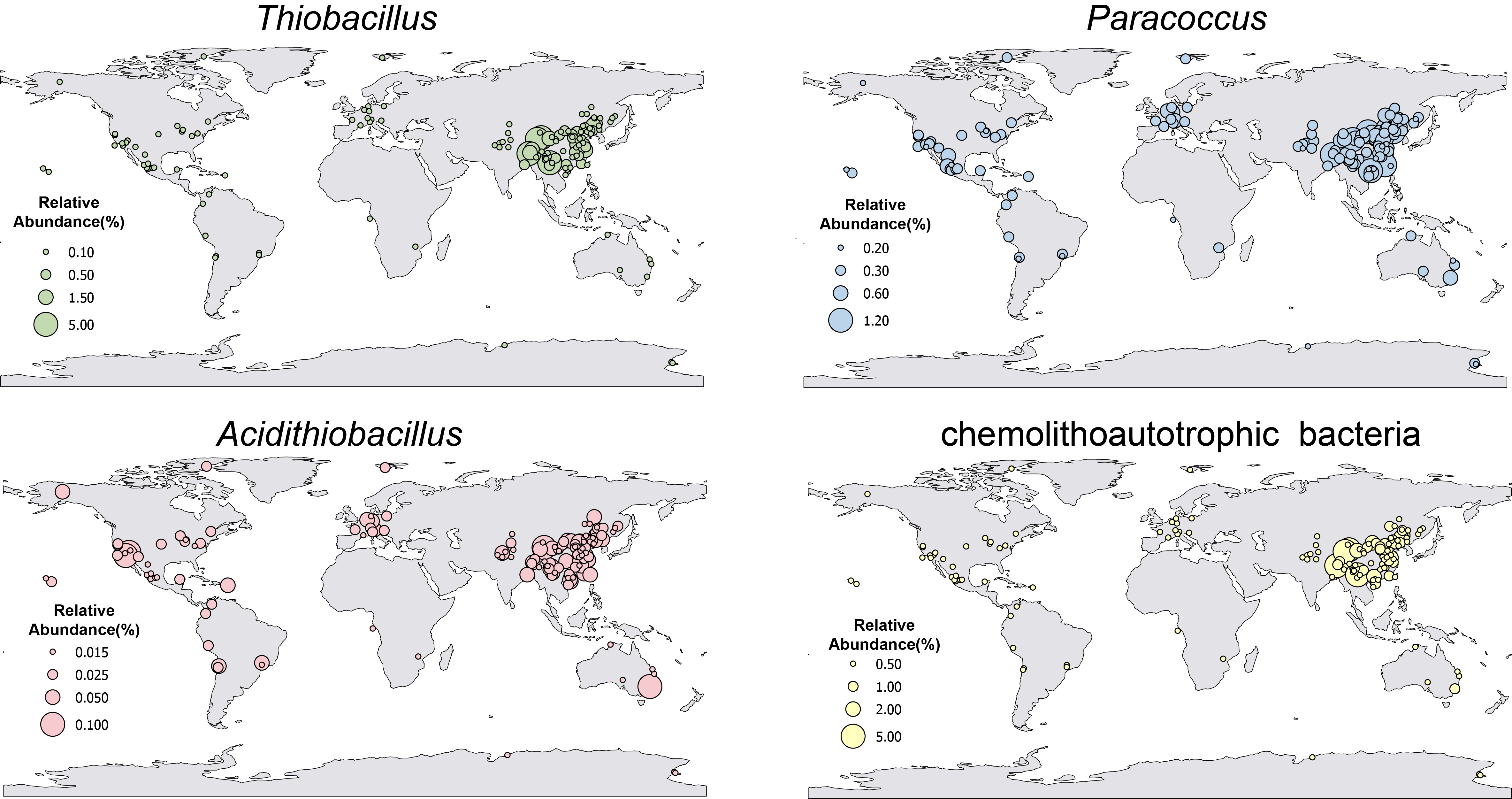


**Figure S12.** Global distribution of the three chemolithoautotrophic bacterial genera—*Thiobacillus*, *Paracoccus* and *Acidithiobacillus*—across global terrestrial soils. Abundance data were derived from metagenomic samples curated from the NCBI database.


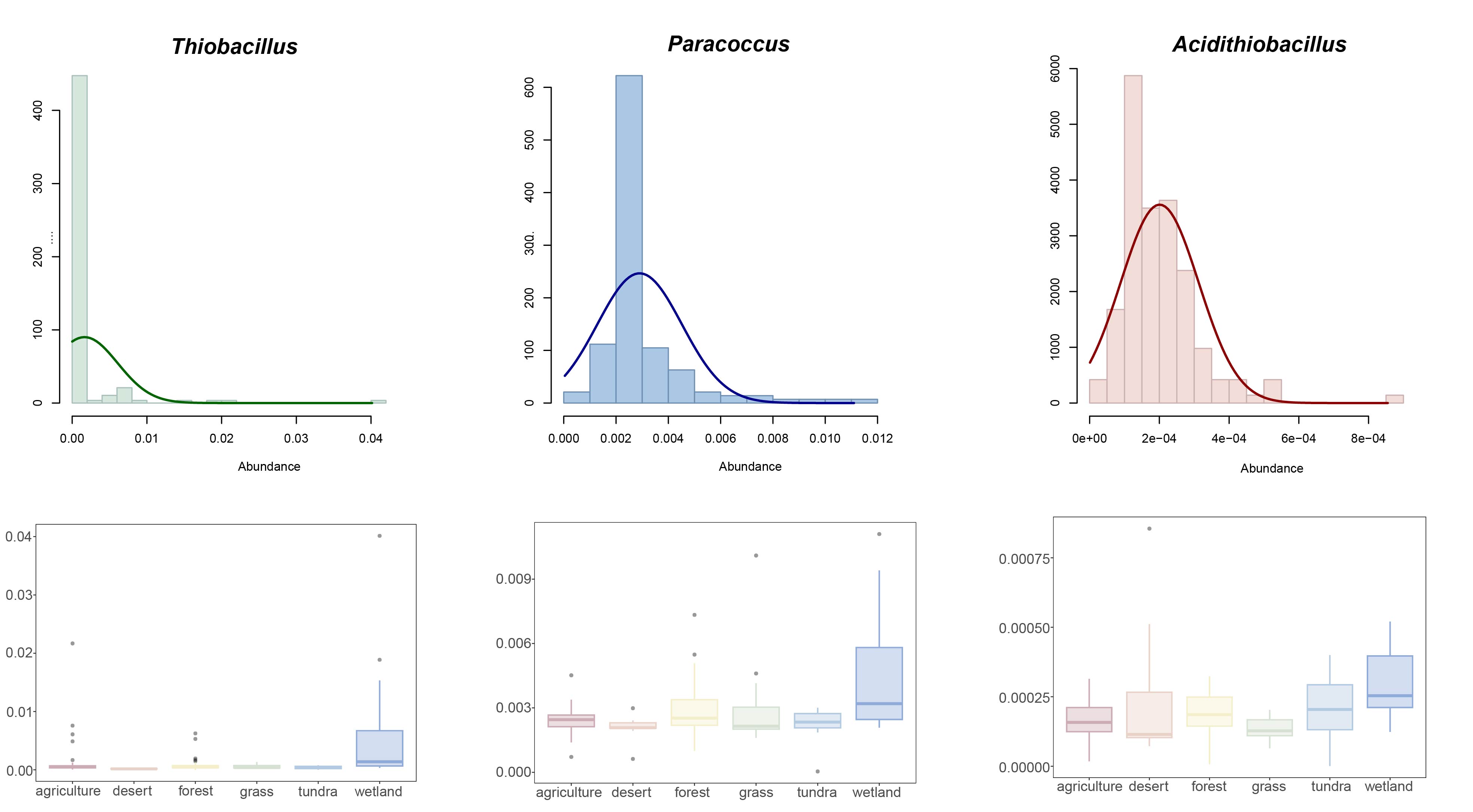


**Figure S13.** Distribution and variability of bacterial abundance across six major habitat types: agriculture, desert, forest, grassland, wetland, and tundra. Boxplots indicate the interquartile range and median values from metagenomic data.


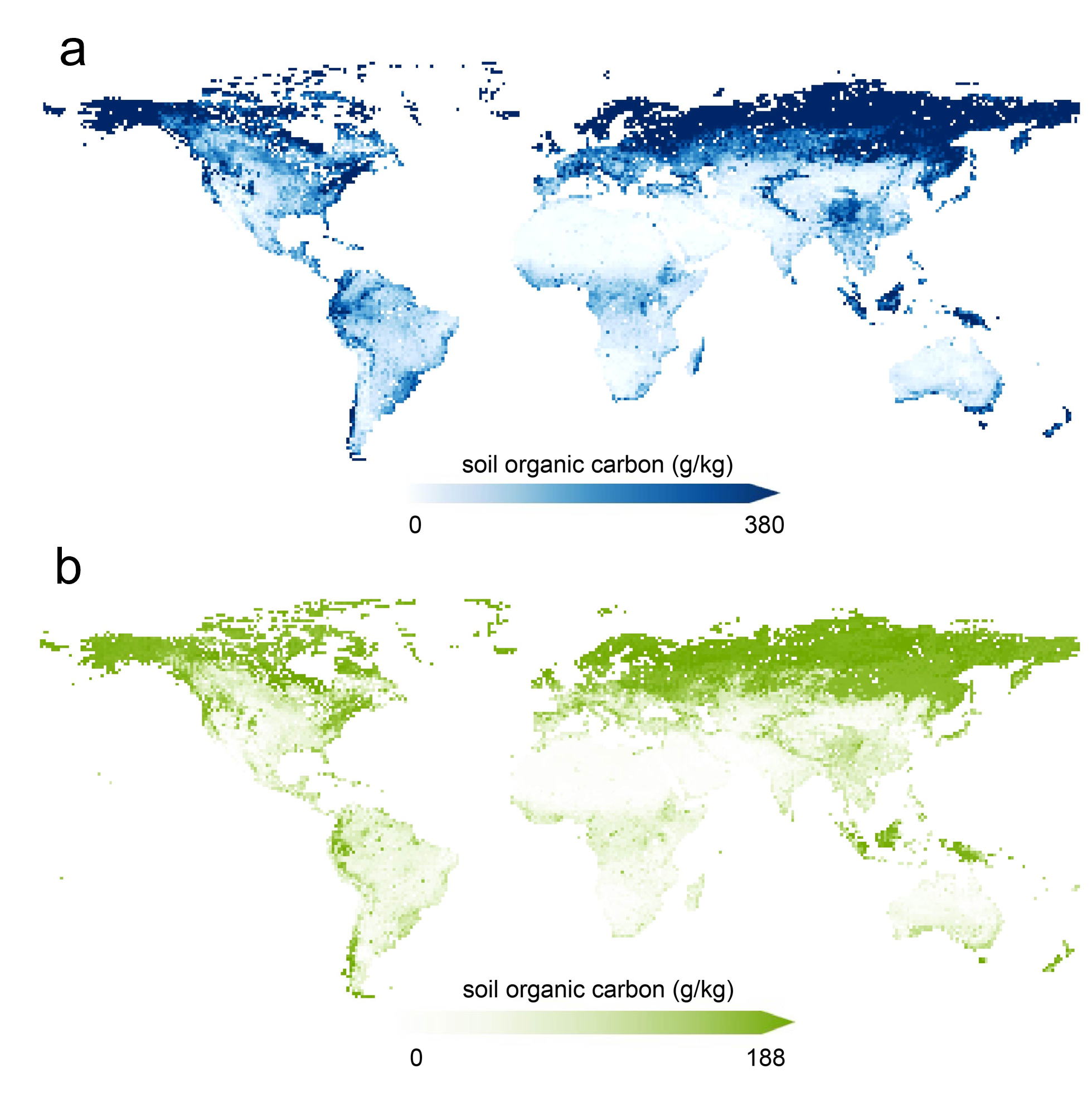


**Figure S14.** Global surface soil organic carbon (SOC) concentration. (a) Mean SOC and (b) standard deviation of SOC concentration in surface soils. Data were obtained from SoilGrids (https://www.isric.org/explore/soilgrids).


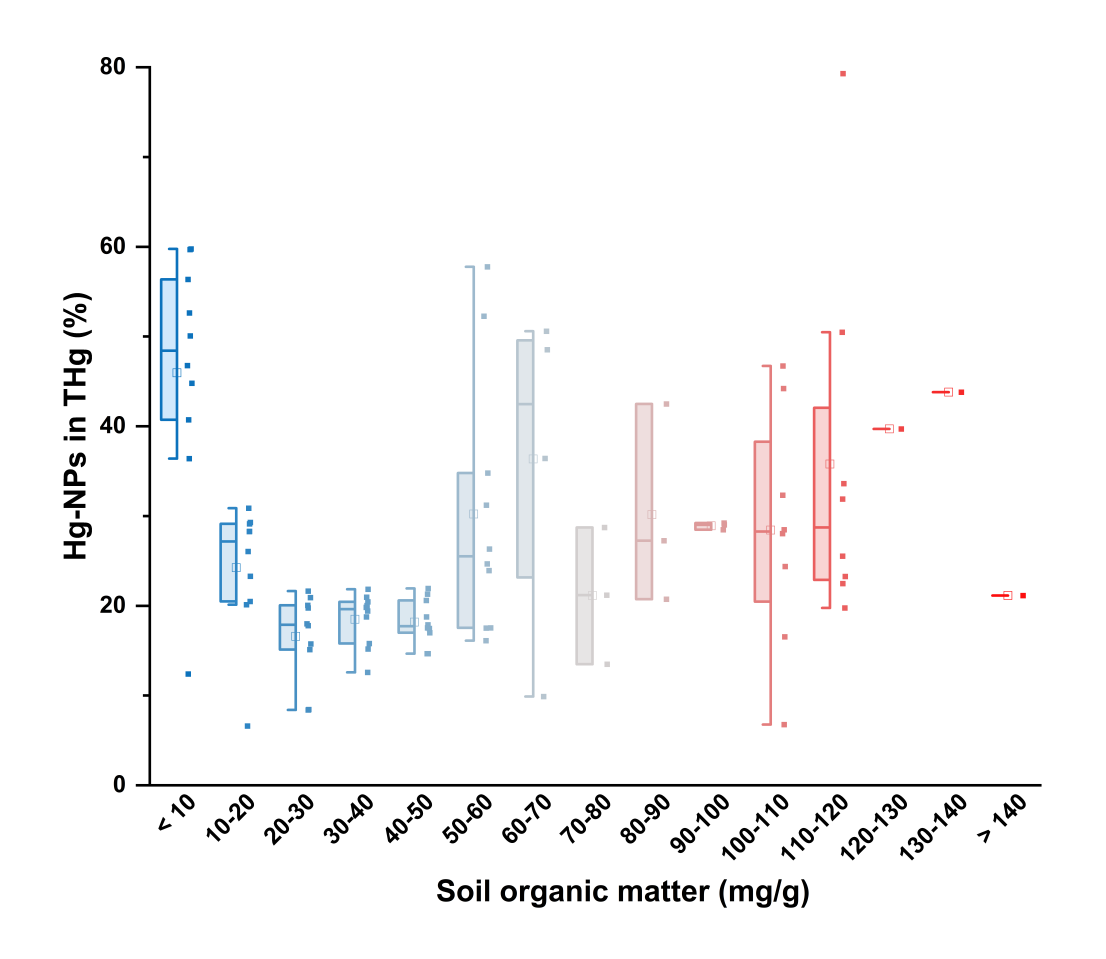


**Figure S15.** Proportion of HgS_NP_ in total soil mercury as a function of SOC. Empirical relationship between SOC content and the fraction of Hg present as HgS_NP_, derived from field measurements and laboratory analyses.


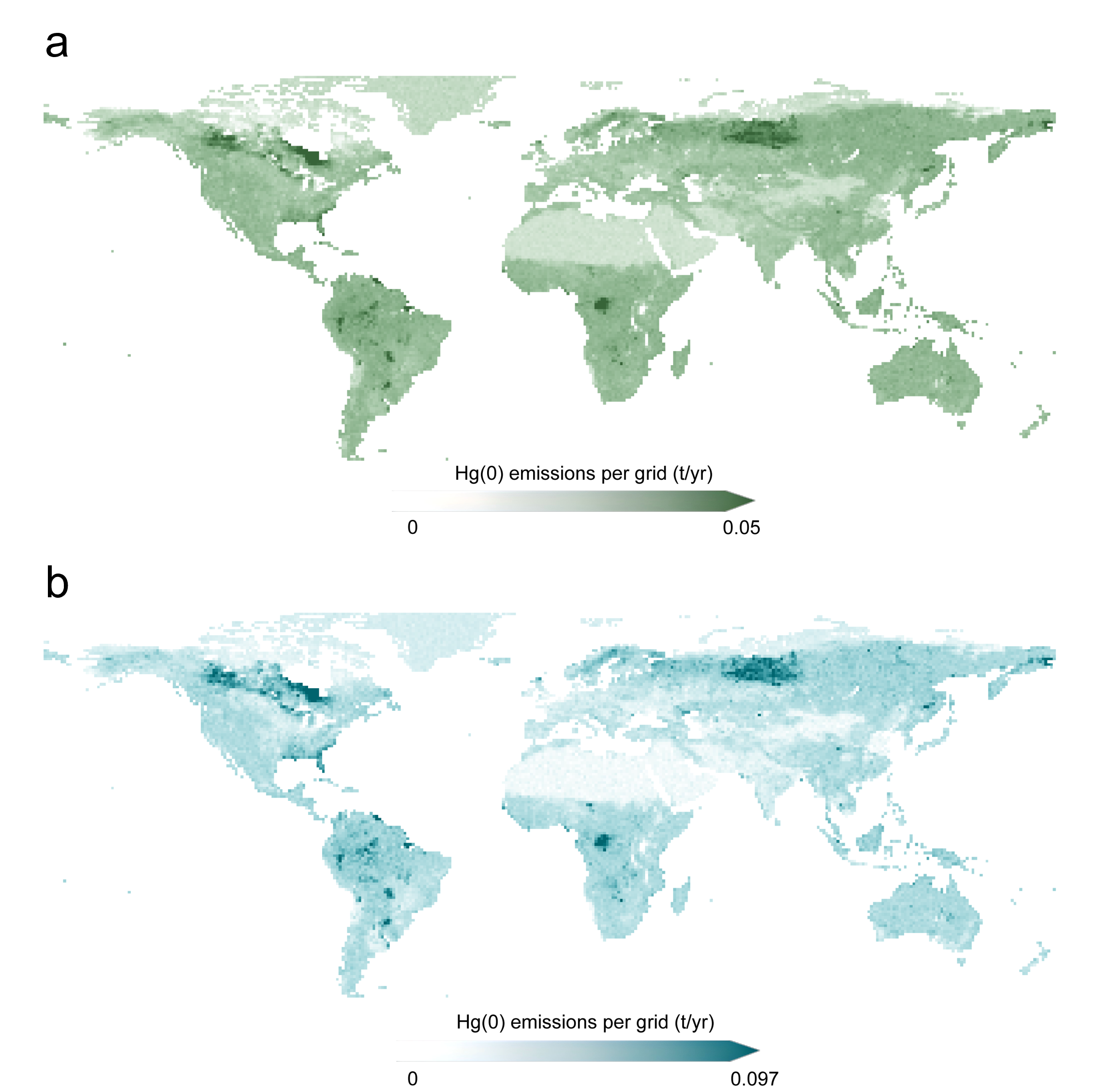


**Figure S16.** Uncertainties of simulated annual Hg^0^ emission due to bacterial reduction of HgS_NP_. (a) Mean and (b) standard deviation of simulated annual Hg^0^ emissions resulting from the reduction of HgS_NP_ by chemolithoautotrophic bacteria. Results are based on 500 Monte Carlo simulations.


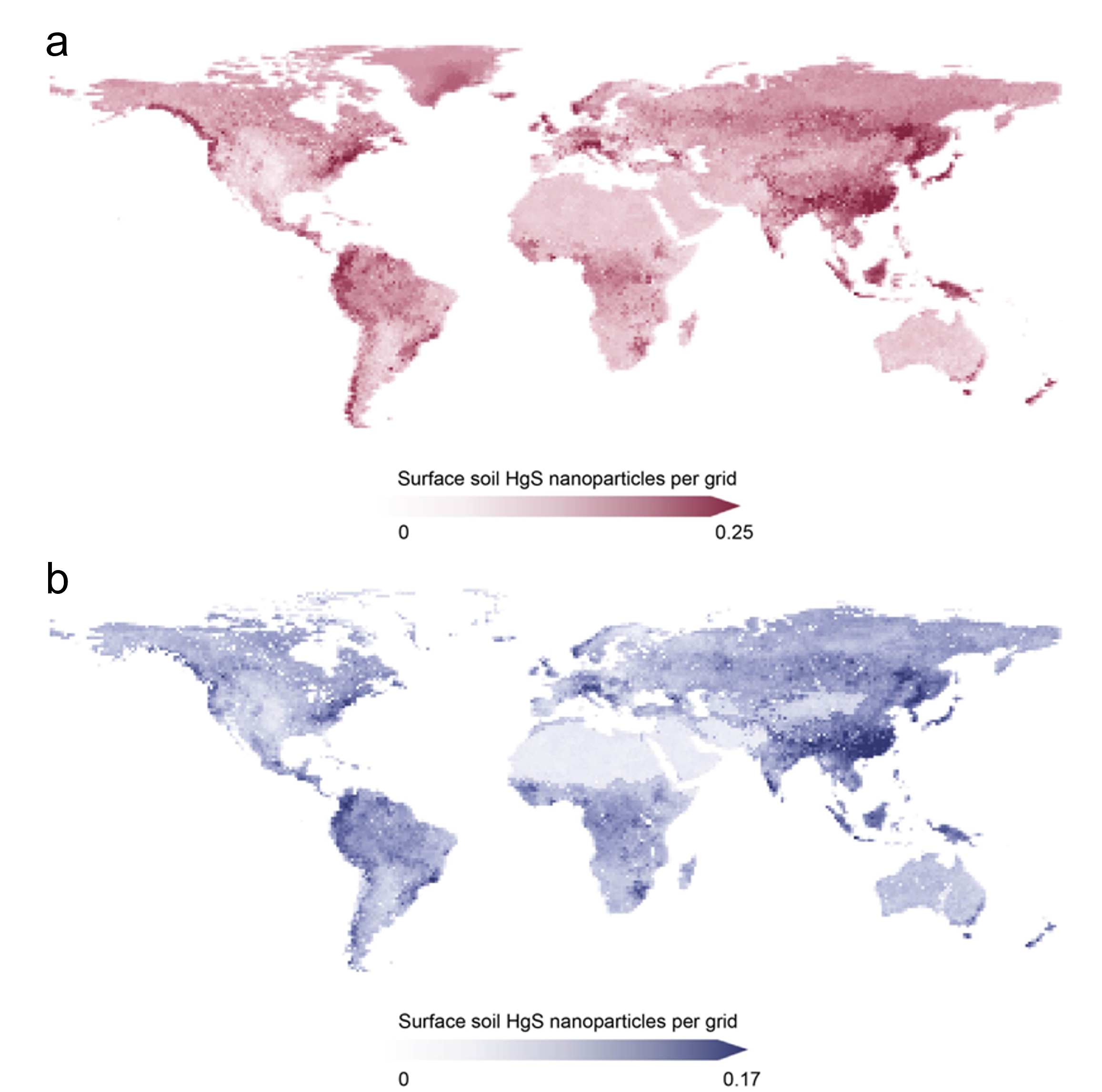


**Figure S17.** Uncertainties of simulated global surface-soil HgS_NP_. (a) Mean and (b) standard deviation of simulated surface-soil HgS_NP_ concentrations obtained from 500 Monte Carlo runs.


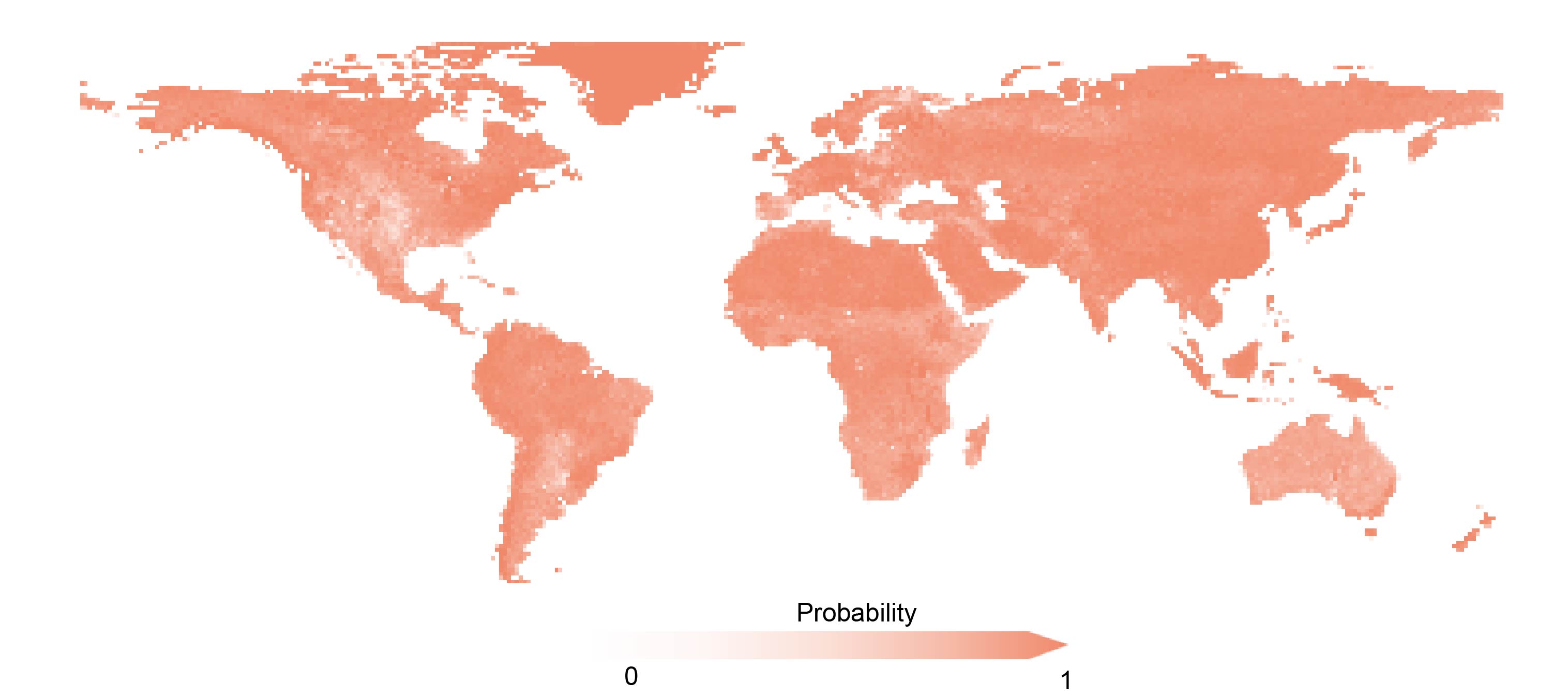


**Figure S18.** Probability of complete conversion of HgS_NP_ to Hg^0^ by chemolithoautotrophic bacteria. Spatial probability that bacterial activity is not limited by HgS_NP_ availability. Estimates are derived from 500 Monte Carlo simulations; values are dimensionless.


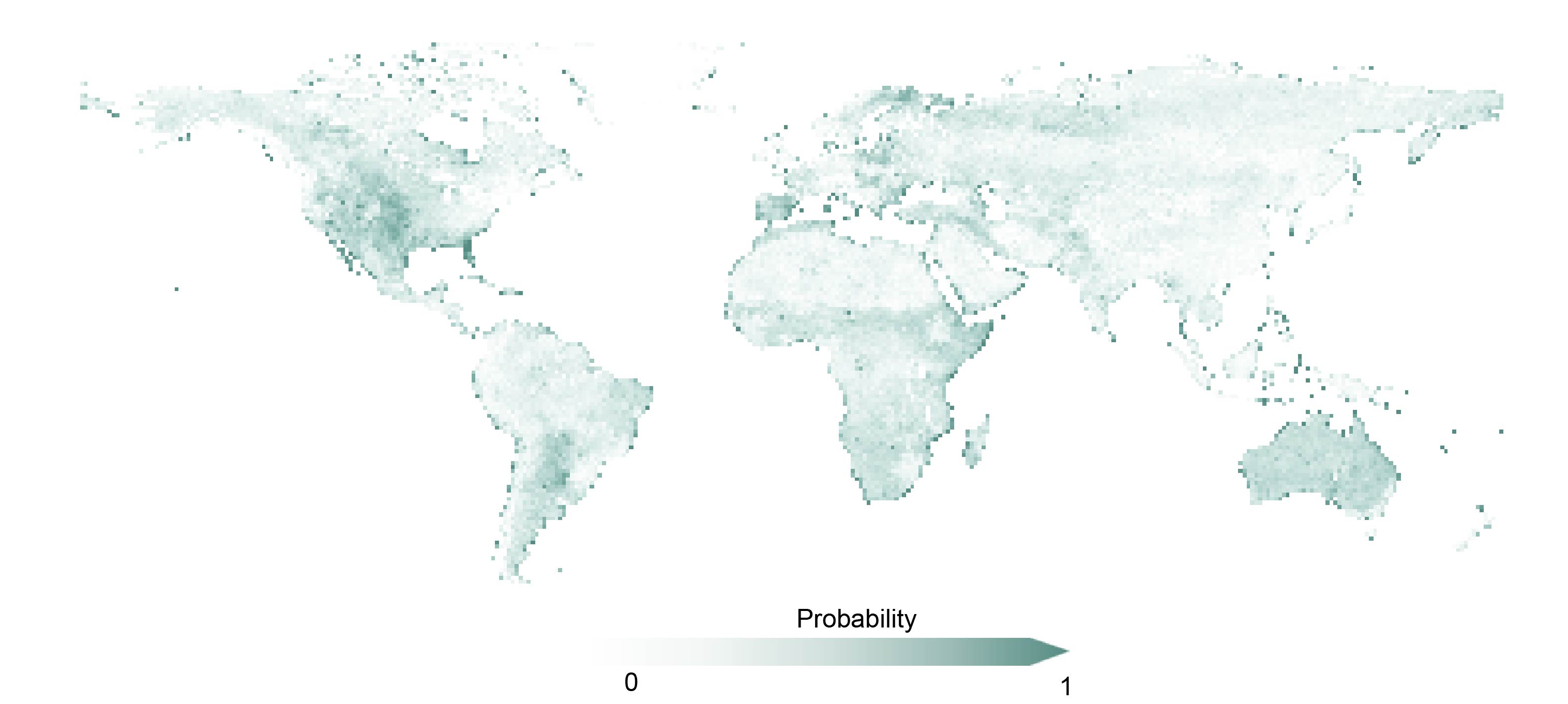


**Figure S19.** Probability of full transformation of surface-soil HgS_NP_. Spatial probability of complete transformation of HgS_NP_ by chemolithoautotrophic bacteria, derived from 500 Monte Carlo realizations; values are dimensionless.


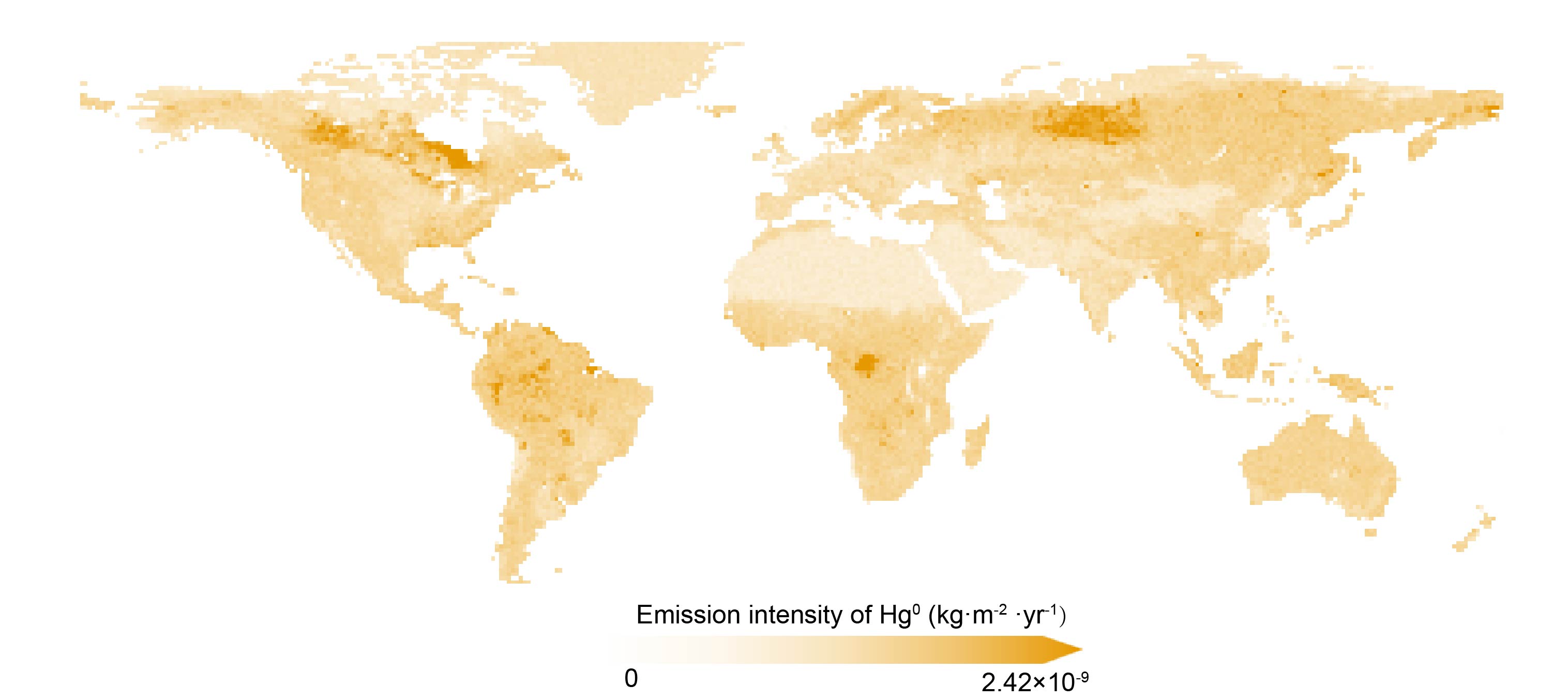


**Figure S20.** Standard deviation of the estimated annual Hg^0^ emission flux from microbial HgS reduction. Spatial distribution of uncertainty (standard deviation) in annual Hg^0^ emission fluxes estimated from bacterial reduction of HgS_NP_. Results based on 500 Monte Carlo simulations.

**Table S1. Primer sequences used for the quantitative real-time reverse transcription–polymerase chain reaction (RT-qPCR) analysis.**

| **Strain** | **Gene** | **Forward primer (5’-3’)** | **Reverse primer (5’-3’)** |
| --- | --- | --- | --- |
| *T. thioparus*  (ATCC 8158) | *sqr* | CAAGCGGTGGATGATGTGGA | TGATAAGGGTTGCGCTCGTT |
|  | *fccB* | TCAGCGTTTCGACTTCGTCA | CCGGTGATGTAGTCGTGCAT |
|  | *soxB* | GCGAGGACATCACGATGGAA | TGGTAATAGGGGTCGGCGTT |
|  | *dsrA* | GAAGGACGAAAACCACCAGC | GGTCGAAGCCCATTTCGTTG |
|  | *dsrB* | AAGGCTCTGAACGACAAGGG | ATTTCGCAGCAAGAGGTGGA |
|  | *aprA* | AAGAAATCCGCCGACAAGGT | ATGCGGTTTTCCATCTGGGT |
|  | *aprB* | CAAGCGGTGGATGATGTGGA | TCCATGTCCTTTTCGCCGTT |
|  | *sat* | CGAACAGATGGGATGGAGCC | GGATCACGGTGTTGGGAACG |
| *P. pantotrophus*  (ATCC 35512) | *sqr* | CAAGCGGTGGATGATGTGGA | TGATAAGGGTTGCGCTCGTT |
|  | *fccB* | TCAGCGTTTCGACTTCGTCA | CCGGTGATGTAGTCGTGCAT |
|  | *soxA* | GGTCTCCATCTTCGGTGACG | ATCTTCAGTTCGGGGCTGTG |
|  | *soxB* | CCATGTCCTGTCCCTTGGTC | GCGAGCCCGAAATCAACATC |
|  | *soxC* | TTTTCCACCAGCCCGTTGAT | TCCTCGATCAACTTCACGCC |
|  | *soxD* | GCACCATCATCAAGCCCAAG | TACACGAGGGGGATGGTCTT |
| *A. ferrooxidans* (ATCC 23270) | *sqr* | CGTCGAGGGCCTCACTAATC | CCTCGACAGGCGGAATGG |
|  | *fccB* | TCAGCGTTTCGACTTCGTCA | CCGGTGATGTAGTCGTGCAT |
|  | *soxB* | CCACACTGACCGAGATGACC | CGCCGTGTTGATAGTAGGGG |
|  | *dsrA* | GAAGGACGAAAACCACCAGC | GGTCGAAGCCCATTTCGTTG |
|  | *dsrB* | AAGGCTCTGAACGACAAGGG | ATTTCGCAGCAAGAGGTGGA |
|  | *aprA* | GTCGAGCAACTGGTGGAAGA | CAGCATCTTGGGGGTGATGT |
|  | *aprB* | GAAGCTGGACAAGGACGGTT | CAGGAATAGCACTCCCAGCA |
|  | *hdr* | TACGAGTGCTGGAAGCAAGG | TGTGAACGCCCAGTTTCTCG |
|  | *sat* | CGAACAGATGGGATGGAGCC | GGATCACGGTGTTGGGAACG |
|  | *merA* | TCCGCAAGTAGCGACGGTAGG | ACCATCGTCAGATAAGGAAAGA |
|  | *iro* | TGGTCGGAGTAAACCCCATT | GCTACCACCTTACAGCACTT |
|  | *coxB* | AACAGGTATGTGGTGGCATC | GCTTGGTGATGGTCTGGATT |
|  | *coxA* | CACTCCCAGCAACATCATCA | TTTGGAGGAAAAGAAGCCCC |
|  | *coxC* | AGGTCCCACATCGCAAAAAT | GGTTATGCCACGCTAATGGA |
|  | *rus* | GGATGCCAGGTAAAATCCGT | CGGGATTTCCATTTCCGAGT |

**Table S2. Proportion of mercury nanoparticles to total mercury according to the literature.**

| **Sampling sites** | **Total mercury**  **(mg kg^-1^)** | **Proportion of mercury nanoparticles**  **to total mercury (%)** | **Reference** |
| --- | --- | --- | --- |
| Guangdong | 0.103 | 22 | Cai et al., 2022 [16] |
| Jiangxi | 0.047 | 19 | Cai et al., 2022 [16] |
| Xinjiang | 0.050 | 14 | Cai et al., 2022 [16] |
| Heilongjiang | 0.023 | 40 | Cai et al., 2022 [16] |
| Chongqing | 0.091 | 7 | Cai et al., 2022 [16] |
| Jiangsu | 0.137 | 9 | Cai et al., 2022 [16] |
| Anhui | 0.082 | 16 | Cai et al., 2022 [16] |
| Hainan | 0.034 | 24 | Cai et al., 2022 [16] |
| Shanxi | 0.027 | 30 | Cai et al., 2022 [16] |
| Shandong | 0.010 | 26 | Cai et al., 2022 [16] |
| Hebei | 0.024 | 20 | Cai et al., 2022 [16] |
| Guizhou-1 | 45.7 | 10 | Cai et al., 2022 [16] |
| Guizhou-2 | 357.1 | 3 | Cai et al., 2022 [16] |
| Guizhou-3 | 129 | 48 | Wang et al., 2020 [17] |
| Wuchuan | 3.73 | 56 | Wang et al., 2022 [18] |
| Guizhou-4 | 44 | 50 | Manceau et al., 2018 [19] |
| Guizhou-5 | 88 | 44 | Manceau et al., 2018 [19] |
| Guizhou-6 | 107 | 64 | Manceau et al., 2018 [19] |
| Nevada | 16.85-60.9 | 55 | Manceau et al., 2018 [20] |

**Table S3. Quality assurance/quality control (QA/QC) data for the analysis of dissolved Hg, total Hg and Hg^0^.**

| Analysis | Method | Method detection limit | Method blank | Relative percent difference *^a^* | Recovery of matrix spike *^b^* |
| --- | --- | --- | --- | --- | --- |
| Dissolved Hg | Literature method based on hydride generation atomic fluorescence spectrometry [21] | 0.20 μg L^−1^ | 0.07 ± 0.05 μg L^−1^ (n = 10) | 4.2 ± 2.9%  (n = 5 pairs) | 95.7 ± 5.5%  (n = 10) |
| Total Hg | EPA Method 1631 [2] | 0.20 ng L^−1^ | 0.21 ± 0.05 ng L^−1^ (n = 10) | 3.2 ± 2.9%  (n = 5 pairs) | 97.2 ± 8.4%  (n = 10) |
| Hg^0^ | Literature method based on reduction-purge-trapping and CVAFS [2,3,22] | 0.20 ng L^−1^ | 0.13 ± 0.04 ng L^−1^ (n = 8) | 4.8 ± 4.1%  (n = 5 pairs) | n.d. ^c^ |

*^a^* Relative percent difference was calculated from the matrix spike and matrix spike duplicate results following the calculational procedure in EPA Method 1631.

*^b^* Dissolved Hg and total Hg were spiked using Hg standard solution (VHG-PHGN, o2si Smart Solutions, USA).

^c^ n.d. = not determined.

**REFERENCES**

1. Zhang T, Kim B, Levard C *et al.* Methylation of Mercury by Bacteria Exposed to Dissolved, Nanoparticulate, and Microparticulate Mercuric Sulfides. *Environ Sci Technol* 2012;**46**:6950–8.

2. *Method 1631, Revision D: Mercury in Water by Oxidation, Purge and Trap, and Cold Vapor Atomic Fluorescence Spectroscopy*. U.S. Environmental Protection Agency, 2002.

3. Sanz-Sáez I, Pereira-García C, Bravo AG *et al.* Prevalence of Heterotrophic Methylmercury Detoxifying Bacteria across Oceanic Regions. *Environ Sci Technol* 2022;**56**:3452–61.

4. Abraham MJ, Murtola T, Schulz R *et al.* GROMACS: High performance molecular simulations through multi-level parallelism from laptops to supercomputers. *SoftwareX* 2015;**1–2**:19–25.

5. Zhang D, Li X, Wu Y *et al.* Microbe-driven elemental cycling enables microbial adaptation to deep-sea ferromanganese nodule sediment fields. *Microbiome* 2023;**11**:160.

6. Grote F, Lyubartsev AP. Optimization of Slipids Force Field Parameters Describing Headgroups of Phospholipids. *J Phys Chem B* 2020;**124**:8784–93.

7. Rappe AK, Casewit CJ, Colwell KS *et al.* UFF, a full periodic table force field for molecular mechanics and molecular dynamics simulations. *J Am Chem Soc* 1992;**114**:10024–35.

8. Berendsen HJC, Postma JPM, Van Gunsteren WF *et al.* Molecular dynamics with coupling to an external bath. *J Chem Phys* 1984;**81**:3684–90.

9. Bussi G, Donadio D, Parrinello M. Canonical sampling through velocity rescaling. *J Chem Phys* 2007;**126**:014101.

10. Darden T, York D, Pedersen L. Particle mesh Ewald: An *N* ⋅log( *N* ) method for Ewald sums in large systems. *J Chem Phys* 1993;**98**:10089–92.

11. Yang Y, Xiao P, Feng X *et al.* Accuracy assessment of seven global land cover datasets over China. *ISPRS J Photogramm Remote Sens* 2017;**125**:156–73.

12. Liu Y-R, Guo L, Yang Z *et al.* Multidimensional Drivers of Mercury Distribution in Global Surface Soils: Insights from a Global Standardized Field Survey. *Environ Sci Technol* 2023;**57**:12442–52.

13. Chen S, Zhou Y, Chen Y *et al.* fastp: an ultra-fast all-in-one FASTQ preprocessor. *Bioinformatics* 2018;**34**:i884–90.

14. Ewels P, Magnusson M, Lundin S *et al.* MultiQC: summarize analysis results for multiple tools and samples in a single report. *Bioinformatics* 2016;**32**:3047–8.

15. Wood DE, Lu J, Langmead B. Improved metagenomic analysis with Kraken 2. *Genome Biol* 2019;**20**:257.

16. Cai W, Wang Y, Feng Y *et al.* Extraction and Quantification of Nanoparticulate Mercury in Natural Soils. *Environ Sci Technol* 2022;**56**:1763–70.

17. Wang J, Shaheen SM, Anderson CWN *et al.* Nanoactivated Carbon Reduces Mercury Mobility and Uptake by Oryza sativa L: Mechanistic Investigation Using Spectroscopic and Microscopic Techniques. *Environ Sci Technol* 2020;**54**:2698–706.

18. Wang J, Man Y, Yin R *et al.* Isotopic and Spectroscopic Investigation of Mercury Accumulation in Houttuynia cordata Colonizing Historically Contaminated Soil. *Environ Sci Technol* 2022;**56**:7997–8007.

19. Manceau A, Wang J, Rovezzi M *et al.* Biogenesis of Mercury-Sulfur Nanoparticles in Plant Leaves from Atmospheric Gaseous Mercury. *Environ Sci Technol* 2018;**52**:3935–48.

20. Manceau A, Merkulova M, Murdzek M *et al.* Chemical Forms of Mercury in Pyrite: Implications for Predicting Mercury Releases in Acid Mine Drainage Settings. *Environ Sci Technol* 2018;**52**:10286–96.

21. Dong X, Ma LQ, Zhu Y *et al.* Mechanistic investigation of mercury sorption by Brazilian pepper biochars of different pyrolytic temperatures based on X-ray photoelectron spectroscopy and flow calorimetry. *Environ Sci Technol* 2013;**47**:12156–64.

22. Zheng W, Hintelmann H. Nuclear field shift effect in isotope fractionation of mercury during abiotic reduction in the absence of light. *J Phys Chem A* 2010;**114**:4238–45.
